# Regiospecific Photochemical Synthesis of Methylchrysenes

**DOI:** 10.3390/molecules28010237

**Published:** 2022-12-28

**Authors:** Thomas Böhme, Mari Egeland, Marianne Lorentzen, Mohamed F. Mady, Michelle F. Solbakk, Krister S. Sæbø, Kåre B. Jørgensen

**Affiliations:** 1 Department of Chemistry, Bioscience and Environmental Engineering, University of Stavanger, P.O. Box 8600, N-4036 Stavanger, Norway; 2 Department of Chemistry and Earth Sciences, College of Arts and Sciences, Qatar University, Doha P.O. Box 2713, Qatar

**Keywords:** polycyclic aromatic hydrocarbon, Mallory reaction, oxidative 6π-electrocyclization, eliminative photochemical cyclization, formylation, methylated PAH, PAH metabolite, oxidation

## Abstract

Methylated polycyclic aromatic hydrocarbons (PAHs) are suspected to be some of the toxic compounds in crude oil towards marine life and are needed as single compounds for environmental studies. 1-, 3- and 6-methylchrysene (**3a,b,c**) were prepared as single isomers by photochemical cyclization of the corresponding stilbenoids in the Mallory reaction using stoichiometric amounts of iodine in 82-88% yield. 2-methylchrysene (**3d**) was prepared by photochemical cyclization where the regioselectivity was controlled by elimination of an *ortho*-methoxy group under acidic oxygen free conditions in 72% yield. These conditions failed to form 4-methylchrysene from the corresponding stilbenoid. All stilbenoids were made from a common naphthyl Wittig salt and suitably substituted benzaldehydes. We have also demonstrated that methylchrysenes can be oxidized to the corresponding chrysenecarboxylic acids by KMnO_4_ in modest yields.

## 1. Introduction

Polycyclic aromatic hydrocarbons (PAHs) are a group of pollutants of great concern, particularly to the aquatic environment [1,2]. Petrogenic PAHs in nature typically originate from industrial or urban effluents, manmade accidents, and discharge of produced water from offshore oil production [1]. Contrary to pyrogenic PAHs, petrogenic PAHs have a large content of alkylation [3]. The concentration of monomethylated chrysenes is typically 10 times higher than chrysene in crude oil [3,4]. Some species, like Atlantic Haddock, subject to commercially important fisheries, are very sensitive to oil pollution at the egg stage [5]. The toxic effects of PAHs are often caused by their metabolites, and the position of alkylation have impact on these effects [1,6,7]. Alkylation on small PAHs makes them more potent agonists than the mother compounds toward aryl hydrocarbon receptors (AHR receptors) that regulate the PAH metabolism [8].

Further studies on the effect of alkylated PAHs require pure single compounds to elucidate these effects in exposure studies, and the compounds made in this work have already contributed to understanding some of the effects of methylation [7,8,9]. When the work described in this paper began, methylated chrysenes where only available as expensive analytical standards is small amounts, but not in the 0.1–0.5 g quantities desired for various environmental exposure studies.

Substituted chrysenes have been made in a variety of methods like the Diels-Alder reaction [10] and intra-molecular Pd-catalyzed C-H activation [11], but most common is photochemical oxidative cyclization, also known as the Mallory reaction [12,13]. The oxidative photocyclization of stilbenes is catalyzed by iodine, and typically air is bubbled through the solution during irradiation [14]. With the extensive studies of this reaction in the 1960–1980′s one might expect all methylated chrysenes to have been made this way before.

**Scheme 1:**
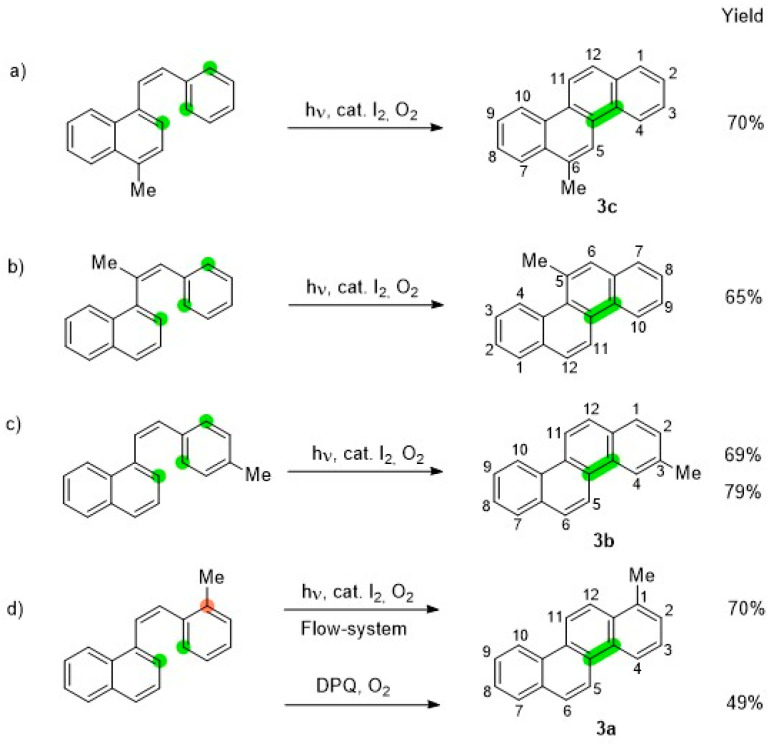
Previous photocyclizations to chrysenes. Green dots mark available positions for photocyclization. Red dots mark blocked positions. Numbering of substituent positions are shown in the products. (**a**) [15], (**b**) [18], (**c**) [19,20], (**d**) [21,22,23].

6-methylchrysene (**3c**) was prepared from photocyclization of styrene attached to a methylated naphthalene in 70% yield [15] (Scheme 1a) and recently by a metathesis reaction [16]. 5-methylchrysene were made in large scale (12 g in 15 L benzene) in 29% yield [17]. Cyclization of 1-(1-phenylprop-1-en-2-yl)naphthalene as 0.02 M in cyclohexane gave 5-methylchrysene in 65% yield after 12 h irradiation [18] (Scheme 1b). No synthesis of 4-methylchrysene nor 2-methylchrysene (**3d**) has ever been published to our best knowledge. 3-Methylchrysene (**3b**) was made by the Mallory reaction in 69% yield after 24 h irradiation [19], and later as a 2.5 g batch in 1 L of cyclohexane in 79% yield after 3 h irradiation with a 400 W high pressure mercury lamp [20] (Scheme 1c). 1-Methylchrysene (**3a**) was made only recently, in a flow system with plugs of air at 100 mg-scale in 89% yield [21,22], while Carrera et al. [23] used a regular immersion well photoreactor with DPQ/air to obtain **3a** in 49% yield (Scheme 1d).

In 1991 Katz’s group developed improved conditions for cyclization of stilbenes [24]. Excluding oxygen prevented degradation by reactive oxygen species formed during the photoreaction. This was possible by using stoichiometric amounts of iodine in degassed solvents with epoxide as a scavenger of the formed HI that will otherwise react with the substrate. We decided to employ these improved conditions in our synthesis of methylated chrysenes and find out if this would give improved yields compared to the literature.

## 2. Results and Discussion

### 2.1. Photochemical Cyclization Using Stoichiometric Amount of I_2_

To obtain a single isomer of a substituted chrysene with the Mallory reaction [12,13] we need both *ortho*-positions to be identical by symmetry, like in the synthesis of **3b** and **3c** (Scheme 1c,d), or one *ortho*-position blocked like in the synthesis of **3a** (Scheme 1d). A substituent in *meta*-position will give two isomers as both *ortho*-positions are available for reaction but giving different products [25].

**Scheme 2:**
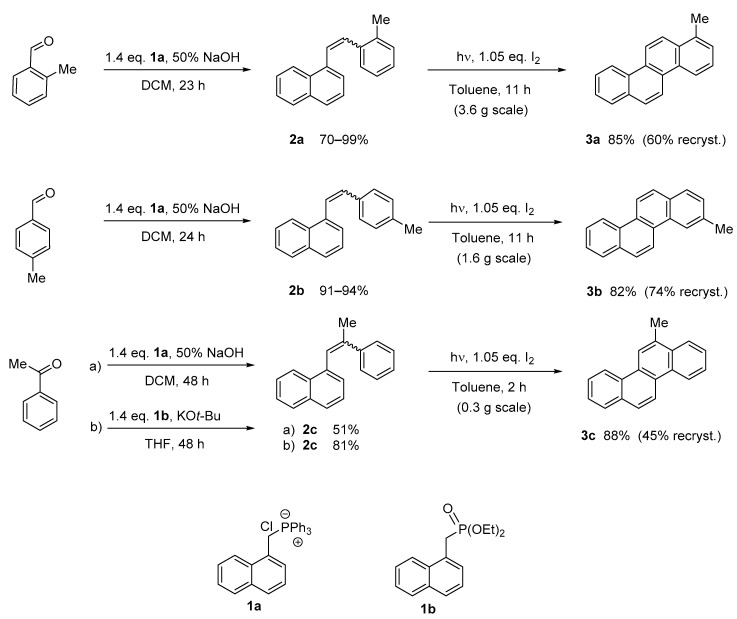
Photocyclization under oxidative conditions.

The stilbenes needed for the photocyclization are readily available through a Wittig reaction. Wittig salt **1a** (Scheme 2) was made by refluxing triphenylphosphine and 1-(chloromethyl)naphthalene in toluene. The product formed a precipitate that was washed with diethyl ether to obtain the pure product in 88% yield. The following Wittig reaction can be performed using an array of different bases. We preferred using a two-phase reaction with 50% aqueous NaOH in dichloromethane [26] at room temperature for practical reasons. Reaction with the suitable benzaldehydes gave stilbenoids **2a** and **2b** in high yields (Scheme 2). The *E*/*Z*-ratio of the stilbenoids have no consequence for the following photocyclization as the double bond isomerize in the process. Close inspection of NMR spectra sometimes allowed determination of the ratio which is then given in the experimental section. We found that the coupling constants for the double bond were about 12 Hz for *Z*-configuration and 15–16 Hz for *E*-configuration. This matches the reported coupling constants of 15.9 Hz (*E*) and 12.1 Hz (*Z*) for styrylnaphthalene [27]. The Wittig-reaction were less reactive with acetophenone giving stilbenoid **2c** (Scheme 2). After 2 days we achieved only 52% yield. The more reactive Wittig-Horner reagent **1b** gave **2c** in 81% yield upon reflux in THF with potassium tert-butoxide as a base.

Finally, the stilbenoids were subjected to the photochemical oxidation with stoichiometric amounts of iodine (Scheme 2). The reactions were followed by TLC, but the disappearing color of iodine in the reaction were also a good indication on completion of the reaction. The photochemical reactions were performed in a 400 W medium pressure mercury lamp in a quartz glass immersion well fitted with a Pyrex filter. The reactions were made in 3–13 mM solution depending on the amount of starting material. Purification with flash chromatography gave **3a–c** in 82–88% yield, better than the results reported for **3b** [20] and 5-methylchrysene [18] using catalytic amounts of I_2_ in a batch reactor. Synthesis of **3a** in a flow reactor gave a similar yield [21,22]. Recrystallization were performed to get melting points and ensure that the compounds intended for toxicology studies were as pure as possible. We intentionally did not synthesize 5-methylchrysene [18] because it was already commercially available, and it is known to be very carcinogenic [28], requiring more strict safety precautions than was available to us.

### 2.2. Photochemical Cyclization under Eliminative Conditions

**Scheme 3:**
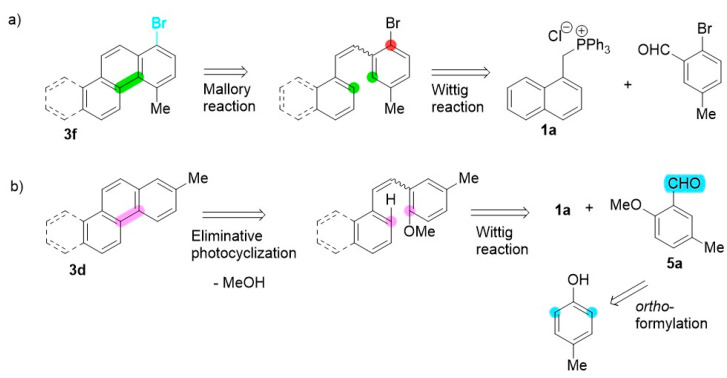
Retrosynthesis: (**a**) Application of a blocking group; (**b**) Eliminative photocyclization.

A synthesis route like in Scheme 2 with a *meta*-substituted stilbenoids would make a mixture of 2- and 4-methylchrysene (**3d,f**) that would be demanding to separate. Olsen and Pruett [29] attempted to control the regioselectivity with a bromine substituent in one *ortho*-position. This worked as a blocking group under regular I_2_, O_2_ conditions (Scheme 3a). Another approach was to eliminate Br in a basic environment without I_2_ nor O_2_ and control the regioselectivity this way. This basic elimination (KO*t*Bu or KOMe in the corresponding alcohol) changed the regioselectivity some but were hampered with significant amounts of regular photocyclization on the unsubstituted *ortho*-position. Regular photochemical cyclization worked better and 1-bromo-4-methylphenanthrene was obtained, but with a significant amount of dehalogenation occurring after the photocyclization. Treating this mixture with LiAlH_4_ gave however 4-methylphenanthrene in 65% yield.

Another approach by Mallory and coworkers [30] used a methoxy group as a controlling group that is eliminated under acidic conditions (A few drops of H_2_SO_4_ in *t*-BuOH/benzene) in the I_2_- and O_2_-free photoreaction (Scheme 3b). They succeed to eliminate *ortho*-methoxy and form 2-methylphenanthrene from the corresponding stilbene in 74% yield. The 4-methylphenanthrene was formed in 53% yield together with 9% 1-methoxy-2-methylphenanthrene. The eliminative reaction was 2–4 times slower than the oxidative reaction, giving 30–175 h irradiation time. 

Considering these options, we decided to try the elimination of a methoxy group, and follow the route outlined in Scheme 3b. Both approaches lacked commercially available starting materials, but the methoxy aldehydes were readily available by the Skattebøl *ortho*-formylation [31,32]. 

**Scheme 4:**
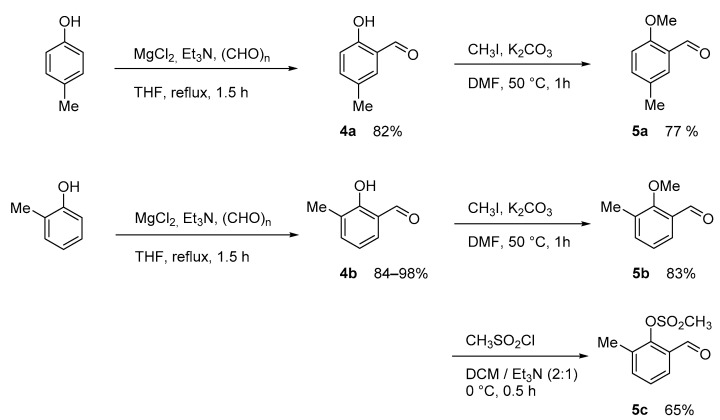
Synthesis of aldehydes.

**Scheme 5:**
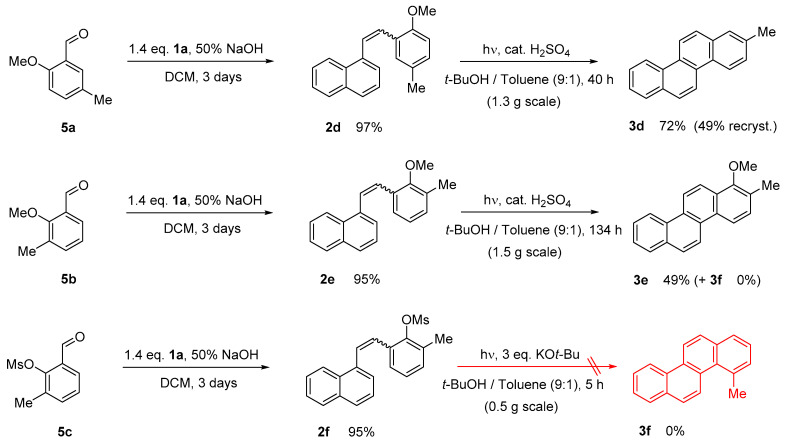
Photocyclization under eliminative conditions.

Formylation of 4-methylfenol, benefiting from the symmetry, gave only aldehyde **4a** (Scheme 4). Aldehyde **4b** was made from 2-methylfenol where only the desired position was available for formylation. After a simple methylation of the hydroxy group these compounds were subjected to the same Wittig reaction (Scheme 5) as the previous aldehydes. Substituting benzene in the original conditions with toluene, the solvent mixture was degassed by ultrasound under N_2_, and kept under a stream of N_2_ during irradiation. After 40 h the starting material was consumed, and 2-methylchrysene (**3d**) could be isolated in 72% yield. Stilbenoid **2e** (Scheme 5) was subjected to the same conditions. Here, the reaction was even slower and was stopped after 134 h with some remaining starting material. The product was isolated in 49% yield but turned out to be the oxidative product **3e**. There was no trace of 4-methylchrysene (**3f**). As 4-methylphenanthrene could be formed this way, although in less amounts than 2-methylphenanthrene, this came as a surprise. Repeated photocyclization of **2e** gave the same result. To make sure nothing was wrong with the procedure nor equipment we made the corresponding stilbene and obtained 4-methylphenanthrene in the same yield as reported [30]. Apparently, the steric hindrance in this reaction [30] increases so much from phenanthrene to chrysene that no **3f** is formed.

As mesyl groups are easily eliminated to form double bonds by base, an attempt on elimination by basic conditions was made. Aldehyde **4b** was protected with a mesyl group (Scheme 4), and stilbenoids **2f** made in the Wittig reaction (Scheme 5). Applying the same base system used for eliminative photocyclization with Br [29], 3 eq. KO*t*Bu in *t*BuOH/toluene, **2f** were irradiated under oxygen free conditions. Unfortunately, the starting material decomposed rather than forming a cyclized product, making a greenish color to the reaction. After 5 h the starting material was decomposed without forming any isolable products.

### 2.3. Direct Oxidation of Methylchrysene 3b

**Scheme 6:**
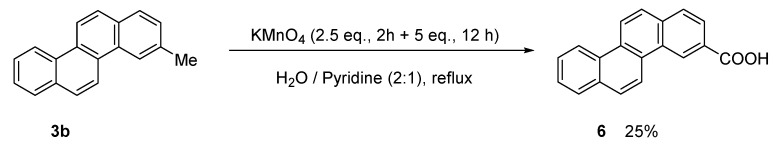
Direct oxidation of 3-methylchrysene (**3b**) to chrysene-3-carboxylic acid (**6**).

One expected metabolite from methylchrysenes is the corresponding carboxylic acids [9]. To provide reference material, experiments were conducted to oxidize **3b** to the corresponding acid. Although toluene has been oxidized to benzoic acid in a wide range of ways, we were unable to find any description of direct oxidation of a methyl group on PAHs larger than naphthalene. Vogel [33] describes oxidation of several substituted toluenes with KMnO_4_, but also describes the oxidation of phenanthrene to biphenyl-2,2’-dicarboxylic acid with hydrogen peroxide. A study on degradation of PAHs by KMnO_4_ found the order of reactivity as benzo[a]pyrene > pyrene > phenanthrene > anthracene > fluoranthene > chrysene [34]. Chrysene being the more stable PAH towards oxidation of the ring system motivated us to attempt oxidation by KMnO_4_. KMnO_4_ slowly degrades in water and several equivalents of reagent are needed [35]. Based on an oxidation procedure in pyridine/water [36], we were after several attempts able to isolate chrysene-3-carboxylic acid (**6**) in 25% yield, when all starting material was consumed (Scheme 6).

## 3. Materials and Methods

### 3.1. General Information

The photochemical reactions were performed in a 400 W medium pressure mercury-lamp in a 2 L quartz immersion well reactor (reaction volume 1.2 L) fitted with a no. 3408 Pyrex glass filter sleeve supplied by Photochemical Reactors Ltd. Silica gel 60A C.C. 40–43 μm from SDS were used for flash chromatography. Melting points were obtained in sealed capillary tubes on a Stuart Scientific melting point apparatus SMP3. NMR-spectra were measured on a Varian Mercury 300 MHz instrument with tetramethylsilane or solvent peak as internal reference (CDCl_3_: 0.0 ppm, 77.0 ppm; CD_3_OD: 3.31 ppm, 49.0 ppm). HRMS analyses were performed on an JMS T100 GC-AccuTOF^TM^ EI-TOF from Jeol.

### 3.2. Synthesis

#### 3.2.1. Synthesis of Wittig-Reagents

*(Naphthalen-1-ylmethyl)triphenylphosphonium chloride* (**1a**)

Triphenylphosphine (28.80 g, 109.8 mmol) and 1-(chloromethyl)naphthalene (17.60 g, 99.6 mmol) was dissolved in toluene (100 mL) and stirred with reflux under nitrogen atmosphere at 120 °C for 2 days. The solvent was removed under reduced pressure, and the solids was washed with diethyl ether (5 × 100 mL) to give 38.43 g (88%) of **1a** as a white solid.

The NMR data were in accordance with those reported by Mousawi et al. [37].

*Diethyl(naphthalen-1-ylmethyl)phosphonate* (**1b**)

The synthesis was inspired by Schwender et al. [38]: 1-(chloromethyl)naphthalene (2.024 g, 11.46 mmol) was stirred in triethylphosphite (4.0 mL, 23 mmol) at 120 °C under N_2_-athmosphere for 68 h. The product was purified by flash chromatography (Petroleum ether: ethyl acetate: isopropanol, 4.5: 4.5: 1). This gave 3.91 g of **1b** as a clear oil (66% pure by ^1^H NMR, approx. 83% yield) containing remains of triethylphosphite. This oil was used without further purification. 

The NMR data were in accordance with those reported [39].

#### 3.2.2. Synthesis of Stilbenes

*General procedure for Wittig-reaction to Stilbenes*

Wittig-salt **1a** (1.2 eq.) and desired aldehyde (1 eq.) in DCM (120 mL) and 50% aq. NaOH (12 mL) was vigorously stirred under N_2_-atmosphere at room temperature until the aldehyde was consumed (1–3 days). The mixture was washed with water (300 mL) and the water phase extracted with DCM (100 mL). The combined DCM-phases were dried with anhydrous MgSO_4_, concentrated under reduced pressure, and purified by flash chromatography (Eluent: Petroleum ether/Ethyl acetate: 19/1) to obtain a mixture of *E*/*Z*-isomers as a viscous oil (94–99% yield). The oil was used in the following photo-cyclization without further purification.

The NMR-spectra of the mixtures were complicated and sometimes also containing a rotamer giving a mix of three NMR-species. NMR-spectra are provided in the supplementary materials. Spectra are partly tabulated for compounds when one isomer can be separated from the NMR-spectra. 

*(E/Z)-1-(2-Methylstyryl)naphthalene* (**2a**)

Wittig-salt **1a** (7.318 g, 16.67 mmol) and 2-methylbenzaldehyde (1.667 g, 13.87 mmol) were reacted for 21 h according to the general procedure to yield **2a** (3.367 g, 99%) as a sticky solid (*E*:*Z* approx. 1:1).

^1^H NMR (300 MHz, CDCl_3_) δ 2.37 (s, 3H), 2.49 (s, 3H), 6.84–7.56 (m, 17 H), 7.72–7.93 (m, 7H), 8.16 (d, *J* = 8.7 Hz, 1H), 8.26 (d, *J* = 7.2 Hz, 1H) ppm. HRMS(EI^+^, TOF) *m*/*z* calcd for C_19_H_16_ [M]^+^ 244.12465, found 244.12482.

*(E/Z)-1-(4-Methylstyryl)naphthalene* (**2b**)

Wittig-salt **1a** (7.288 g, 16.61 mmol) and 4-methylbenzaldehyde (1.656 g, 13.78 mmol) were reacted for 24 h according to the general procedure to yield **2b** (3.163 g, 94%) as a viscous oil (*E*:*Z* approx. 1:1).

NMR spectra are consistent with reported data for (*E*)-**2b** [40]. HRMS (EI^+^, TOF) *m/z* calcd for C_19_H_16_ [M]^+^ 244.12465, found 244.12467.

*(E/Z)-1-(2-Phenylprop-1-en-1-yl)naphthalene* (**2c**)

Wittig-salt **1a** (7.292 g, 16.61 mmol) and acetophenone (1.672 g, 13.92 mmol) were reacted for 48 h according to the general procedure to yield **2c** (1.734 g, 51%) as a viscous oil.

*Alternative procedure:*

A mixture of **1b** (1.36 g, 4.90 mmol) and acetophenone (0.405 g, 3.37 mmol) in dry THF (80 mL) was heated to reflux (oil bath 75 °C) under nitrogen atmosphere. The mixture was then added potassium tert-butoxide (0.600 g, 5.35 mmol) and stirred at reflux for 48 h. The mixture was evaporated onto silica and purified by flash chromatography (Eluent: Petroleum ether/Ethyl acetate: 19/1) to yield **2c** (0.660 g, 81%) as a viscous oil (*E*:*Z* approx. 1:3, based on the Me-signal in ^1^H NMR that is reported to be 2.05 (s, 3H) ppm for (*E*)-**2c** [41], while our minor isomer has the Me-signal at 2.13 (d, *J* = 1.5 Hz, 3H) ppm). 

Major isomer (Z)-**2c**: ^1^H NMR (300 MHz, CDCl_3_) δ 2.36 (d, *J =* 1.5 Hz, 3H), 6.95–6.99 (m, 3H), 7.05–7.54 (m, 9H), 7.62 (d, *J* = 8.6 Hz, 1H), 7.78–7.82 (m, 2H), 8.15 (d, *J* = 6.8 Hz, 1H) ppm; HRMS (EI^+^, TOF) *m/z* calcd for C_19_H_16_ [M]^+^ 244.12465, found 244.12476.

*(E/Z)-1-(2-Methoxy-5-methylstyryl)naphthalene* (**2d**)

Wittigsalt **1a** (6.294 g, 14.33 mmol) and aldehyde **5a** (1.768 g, 11.77 mmol) were reacted for 3 days according to the general procedure to yield **2d** (2.770g, 97%) as a viscous oil (*E*:*Z* approx. 1:3).

Major isomer: (*Z*)-**2d:** ^1^H NMR (300 MHz, CDCl_3_) δ 1.88 (s, 3H), 3.71 (s, 3H), 6.68 (s, 1H), 6.70 (d, *J* = 6.0 Hz, 1H), 6.87 (d, *J* = 8.4 Hz, 1H), 6.97 (d, *J* = 12.2 Hz, 1H), 7.08 (d, *J =* 12.2 Hz, 1H), 7.22-7.38 (m, 1H), 7.44–7.49 (m, 3H), 7.69 (d, *J* = 7.8 hz, 1H), 7.81–7.84 (m, 1H), 8.09–8.12 (m, 1H) ppm; ^13^C NMR (75 MHz, CDCl_3_) δ 20.2, 55.5, 110.4, 124.7, 125.4, 125.7, 125.8, 126.5, 127.1, 127.2, 128.1, 128.3, 128.7, 130.5, 125-130(2C), 131.7(C), 133.6(C), 135.6(C), 155.1(C) ppm; HRMS (EI^+^, TOF) *m/z* calcd for C_20_H_18_O_1_ [M]^+^ 274.13523, fund 274.13530.

*(E/Z)-1-(2-Methoxy-3-methylstyryl)naphthalene* (**2e**)

Wittigsalt **1a** (5.854 g, 13.33 mmol) and aldehyde **5b** (1.686 g, 11.22 mmol) was reacted for 3 days according to the general procedure (Flash eluent Petroleum ether/Ethyl acetate: 19/1) to yield **2e** (2.577 g, 95%) as a viscous oil (*E/Z* = 1:3). 

Major isomer (Z)-**2e**: ^1^H-NMR (300 MHz, CDCl_3_) δ 2.28 (s, 3H), 3.86 (s, 3H), 6.51–6.56 (m, 1H), 6.67 (d, *J* =7.6 Hz, 1H), 6.93 (d, *J* = 7.3 Hz, 1H), 7.05 (d, *J* =12.2 Hz, 1H), 7.13 (d, *J* = 12.2 Hz, 1H), 7.26–7.50 (m, 3H), 7.67–7.72 (m, 2H), 7.81-7.85 (m, 1H), 8.08–8.12 (m, 1H) ppm; ^13^C-NMR (75 MHz, CDCl_3_) δ 16.0, 60.7, 123.2, 124.6, 125.5, 125.9, 126.0, 126.6, 127.4 (2CH), 128.0, 128.4, 128.6, 130.1, 123-131(2C), 131.7(C), 133.6(C), 135.0(C), 156.9(C) ppm; HRMS (EI^+^, TOF) m/z calcd for C_20_H_18_O_1_ [M]^+^ 274.13521, found 274.13490.

*(E/Z)-2-Methyl-6-(2-(naphthalen-1-yl)vinyl)phenyl methanesulfonate* (**2f**)

Wittigsalt **1a** (4.462 g, 10.16 mmol) and aldehyde **5c** (1.779 g, 8.304 mmol) was reacted for 2 h according to the general procedure (DCM (75 mL), 50% aq. NaOH (7.5 mL), flash eluent: Petroleum ether/Ethyl acetate: 1/1) to yield **2f** (2.221 g, 79%) as a viscous oil.

NMR spectra are given in the SI. No single isomer can be tabulated. HRMS(ESI^+^, TOF) *m*/*z* calcd for C_20_H_18_NaO_3_S [M+Na]^+^ 361.08688, found 361.08713.

#### 3.2.3. Photochemical cyclization of stilbenes

*1-Methylchrysene* (**3a**)

The photoreactor was flushed with N_2_ and loaded with stilbene **2a** (3.660g, 14.98 mmol), I_2_ (4.190 g, 16.49 mmol), 1,2-epoxybutane (21 mL, 247 mmol) and degassed toluene (1200 mL). The reaction was stirred under N_2_ atmosphere until all was dissolved, and stirred under UV-irradiation for 11 hrs. The reaction mixture was reduced to half volume under reduced pressure and washed with sat. aq. Na_2_S_2_O_3_ (400 mL). The water phase was extracted with ethyl acetate (250 mL) and the combined organic phases washed with brine (250 mL) and dried over anhydrous MgSO_4_. The product was isolated by flash chromatography (Petroleum ether/ethyl acetate: 19/1) to yield 3.09 g (85%) of **3a**. 

This product was recrystallized from heptane/chloroform (100/70 mL), and the remains once more with heptane/chloroform (50/15 mL) to yield together 2.19 g (60%) of **3a** as white crystals. Melting point: 254–255 °C. Lit. 250.4–254 °C [42].

^1^H NMR (300 MHz, CDCl_3_) δ 2.82 (s, 3H), 7.49 (d, *J* = 6.9 Hz, 1H), 7.57–7.73 (m, 3H), 7.99 (d, *J* = 8.7 Hz, 2H), 8,20 (d, *J* = 9,3 Hz, 1H), 8.66–8.80 (m, 4H) ppm; ^13^C NMR (75MHz, CDCl_3_) δ 19.9(CH_3_), 120.9(CH), 121.4(CH), 121.5(CH), 123.1(CH), 123.3(CH), 126.25(CH), 126.31(CH), 126.6(CH), 127.3(CH), 127.4(CH), 127.8(C), 128.5(CH), 128.6(C), 130.5(C), 130.6(C), 131.1(C), 132.0(C), 134.9(C) ppm.

The NMR spectra were in accordance with those reported by Lutnæs and Johansen [43]. 

*3-Methylchrysene* (**3b**)

The photoreactor was flushed with N_2_ and loaded with stilbene **2b** (1.631g, 6.675 mmol), I_2_ (1.894 g, 7.462 mmol), 1,2-epoxybutane (14.45 g, 200.4 mmol) and degassed toluene (1200 mL). The reaction was stirred under N_2_ atmosphere until all was dissolved, and stirred under UV-irradiation for 11 hrs. The reaction mixture was reduced to 400 mL under reduced pressure and washed with sat. aq. Na_2_S_2_O_3_ (300 mL). The water phase was extracted with EtOAc (200 mL) and the combined organic phases washed with brine (200 mL) and dried over anhydrous MgSO_4_. The product was isolated by flash chromatography (Petroleum ether/ethyl acetate 19:1) to yield 1.324 g (82%) of **3b**. 

Product from this batch and 2 pilot batches were recrystallized together from methanol/chloroform, and the remains once more with methanol/chloroform to yield a total of 1.688g (74%) of **3b** as white crystals. Melting point: 174.0–174.7 °C. Lit: 173–175 °C [11]. 

^1^H NMR (400 MHz, CDCl_3_) δ 2.67 (s, 3H), 7.48 (dd, *J* = 8.1, 1.2 Hz, 1H), 7.63 (ddd, *J* = 7.4, 7.0, 1.2 Hz, 1H), 7.71 (ddd, *J* = 7.6, 6.9, 1.4 Hz, 1H), 7.90 (d, *J* = 8.1Hz, 1H), 7.97–8.01 (m, 3H), 8.58 (s, 1H), 8.66 (d, *J* = 9.1 Hz, 1H), 8.72 (d, *J* = 9.1 Hz, 1H), 8.78 (d, *J* = 8.3 Hz, 1H) ppm; ^13^C NMR (100 MHz, CDCl_3_) δ 22.3(CH3), 120.3(CH), 121.3(CH), 122.7(CH), 123.2(CH), 126.3(CH), 126.6(CH), 127.0(CH), 127.1(CH), 127.8(C), 128.26(CH), 128.34(C), 128.4(CH), 128.5(CH), 130.2(C), 130.61(C), 130.64(C), 132.1(C), 136.4(C)ppm. 

The NMR spectra were in accordance with those reported by Lutnæs and Johansen [43].

*6-Methylchrysene* (**3c**)

The photoreactor was flushed with N_2_ and loaded with stilbene **2c** (0.3150 g, 1.289 mmol), I_2_ (0.3600 g, 1.418 mmol), 1,2-epoxybutane (2.806 g, 38.91 mmol) and degassed toluene (400 mL). The reaction was stirred under N_2_ atmosphere until all was dissolved, and stirred under UV-irradiation for 2 hrs. The reaction mixture was reduced to 200 mL under reduced pressure and washed with sat. aq. Na_2_S_2_O_3_ (150 mL). The water phase was extracted with ethyl acetate (100 mL) and the combined organic phases was washed with brine (100 mL) and dried over anhydrous MgSO_4_. The product was isolated by flash chromatography (Petroleum ether/ethyl acetate 19/1) to yield 0.274 g (88%) of **3c**. 

The product was recrystallized from methanol/chloroform in two rounds to yield 0.140 g (44%) of **3c** as white crystals. Melting point: 158.7–160.1 °C. Lit. 159–161 °C [44].

^1^H NMR (300 MHz, CDCl_3_) δ 2.89 (s, 3H), 7.61–7.71 (m, 4H), 7.943-7.98 (m, 2H), 8.16 (d, *J* = 7.9 Hz, 1H), 8,56 (s, 1H), 8.70 (d, *J* = 9.1 Hz, 1H), 8.77–8.82 (m, 2H) ppm; ^13^C NMR (75MHz, CDCl_3_) δ 20.6(CH3), 121.1(CH), 121.5(CH), 123.1(CH), 123.6(CH), 124.7(CH), 126.2(CH), 126.27(CH), 126.31(CH), 126.4(CH), 126.5(CH), 127.3(C), 128.0(C), 128.5(CH), 130.2(C), 130.6(C), 131.9(C), 132.2(C), 133.1(C) ppm. 

The NMR-spectra were in accordance with those reported by Lutnæs and Johansen [43].

*2-Methylchrysene* (**3d**)

Stilbene **2d** (1.303 g, 4.750 mmol) was dissolved in a degassed 9:1 mixture of *t*-butanol/toluene (1200 mL) under N_2_ in the photoreactor and added 4 drops of conc. H_2_SO_4_. The mixture was irradiated for 40 hrs (followed by TLC) and concentrated under reduced pressure. The crude was purified by column chromatography (Petroleum ether/Ethyl acetate 9/1) to yield 0.828 g (72%) of **3d** as a pale-yellow solid. 

Recrystallization from chloroform/methanol gave 0.559 g (49%) of **3d** as white crystals. Melting point: 230–231 °C. Lit. 229–230 °C [45].

^1^H NMR (300 MHz, CDCl_3_) δ 2.56 (s, 3H), 7.50 (d, *J* = 8.5 Hz, 1H), 7.56–7.69 (m, 2H), 7.73 (s, 1H), 7.87–7.96 (m, 3H), 8.61–8.66 (m, 3H), 8.73 (d, *J* = 8.3 Hz, 1H) ppm; ^13^C NMR (75 MHz, CDCl_3_) δ 21.5(CH_3_), 121.1(2CH), 123.00(CH), 123.01(CH), 126.1(CH), 126.6(CH), 126.9(CH), 127.2(CH), 127.6(C), 127.9(CH), 128.2(C), 128.5(CH), 128.6(CH), 130.6(C), 131.9(C), 132.3(C), 136.1(C) ppm.

The NMR-spectra were in accordance with those reported by Lutnæs and Johansen [43].

*1-Methoxy-2-methylhrysene* (**3e**)

Stilbene **2e** (1.401 g, 5.106 mmol) was dissolved in a degassed 9:1 mixture of *t*-butanol/toluene (1200 mL). The mixture was added 4 drops of conc. H_2_SO_4_ and loaded into the photoreactor under N_2_ atmosphere. After irradiation for 134 h, the mixture was concentrated under reduced pressure and purified by flash chromatography (Petroleum ether/Ethyl acetate: 9/1) to afford 0.674 g (49%) of **5a** as a colorless solid.

Melting point of recrystallized compound (CHCl_3_/methanol): 191–192 °C. 

^1^H NMR (300 MHz, CDCl_3_) δ 2.53 (s, 3H), 3.97 (s, 3H), 7.51 (d, *J =* 8.5 Hz, 1H), 7.59–7.71 (m, 2H), 7.94–7.98 (m, 2H), 8.31 (d, *J* = 9.3 Hz, 1H), 8.44 (d, *J =* 8.6 Hz, 1H), 8.64 (d, *J* = 9.0 Hz, 1H), 8.72 (d, *J =* 9.3 Hz, 1H), 8.77 (d, *J =* 8.3 Hz, 1H) ppm; ^13^C NMR (75 MHz, CDCl_3_) δ 15.9(CH_3_), 61.3(OCH_3_), 119.0(C), 121.0(CH), 121.2(CH), 121.3(CH), 123.1(CH), 126.2(CH), 126.59(C), 126.63(CH) 127.1(C), 127.3(CH), 127.7(C), 128.4(C), 128.5(CH), 129.9(CH), 130.5(C), 130.6(C), 132.0(C), 154.3(C); HRMS (EI^+^, TOF) *m*/*z* calcd for C_20_H_16_O [M]^+^ 272.11957, found 272.11972.

*Attempted photocyclization of stilbene* **2f**

Stilbene **2f** (0.532 g, 1.57 mmol) was dissolved in degassed *t*-Butanol/toluene (9:1, 300 mL) together with potassium *t*-butoxide (0.490 g, 4.37 mmol) under N_2_ atmosphere. The mixture was irradiated for 5 hrs. The solution got a greenish cast to it, and starting material was decomposing without any product being formed.

#### 3.2.4. Formylation of phenoles

*2-Hydroxy-5-methylbenzaldehyde* (**4a**)

Following the description of Hansen and Skattebøl [31], water free MgCl_2_ (2.973 g, 31.22 mmol) and paraformaldehyde (4.701 g, 156.5 mmol) was dissolved in dry THF (100 mL) under N_2_ atmosphere. Triethylamine (10.5 mL) was added dropwise under stirring. After 10 min. 4-methylfenol (2.170 g, 20.06 mmol) was added dropwise. The mixture was refluxed in an oil bath at 75 °C for 1.5 h. After reaching room temperature the mixture was transferred to a separating funnel with diethyl ether (35 mL), and the organic phase washed with 1 M HCl (3 × 35 mL), water (2 × 35 mL) and brine (35 mL). The organic phase was dried over anhydrous MgSO_4_ and concentrated under reduced pressure to afford 2.226 g (82%) of **4a** as a tick oil that was used without further purification.

^1^H NMR and ^13^C NMR were in accordance with the description of Batt and Nayak [46].

*2-Hydroxy-3-methylbenzaldehyde* (**4b**)

Following the description of Hofsløkken and Skattebøl [32], water free MgCl_2_ (2.956 g, 31.04 mmol) and paraformaldehyde (4.622 g, 155.0 mmol) was dissolved in dry THF (100 mL) under N_2_ atmosphere. Triethylamine (10.5 mL) was added dropwise under stirring. After 10 min. 2-methylphenol (1.995 g, 18.44 mmol) was added. The mixture was refluxed in an oil bath at 75 °C for 1.5 h. After reaching room temperature the mixture was transferred to a separation funnel with diethyl ether (35 mL), and the organic phase was washed with 1M HCl (3 × 35 mL), water (2 × 35 mL) and brine (35 mL) The organic phase was dried over anhydrous MgSO_4_ and concentrated under reduced pressure to afford 2.456 g (98%) of **4b** as a tick oil that was used without further purification.

^1^H NMR and ^13^C NMR were in accordance with the description by Aspinall et al. [47].

#### 3.2.5. Protection of Hydroxybenzaldehydes

*2-Methoxy-5-methylbenzaldehyde* (**5a**)

Aldehyde **4a** (2.226 g, 16,35 mmol) was dissolved DMF (9 mL) in an oil bath at 50 °C. The mixture was added K_2_CO_3_ (2.733 g, 19.77 mmol) and iodomethane (1.40 mL, 22.5 mmol), and stirred for 1 h. Upon reaching room temperature, water (20 mL) was added, and pH adjusted to 7 with 1 M HCl before extraction with ethyl acetate 2 × 10 mL). The combined organic phases were washed with brine (10 mL), dried over anhydrous MgSO_4_ and concentrated under reduced pressure. The remains were purified by flash chromatography (Petroleum ether:/Ethyl acetate 9/1) to afford 1.896 g (77%) of **5a** as an oil. 

^1^H NMR (300 MHz, CDCl_3_) δ 2.31 (s, 3H), 3.90 (s, 3H), 6.92 (d, *J =* 8.5 Hz, 1H), 7.36 (dd, *J =* 8.5, 1.9 Hz, 1H), 7.63 (d, *J =* 1.9 Hz, 1H), 10.45 (s, 1H); ^13^C NMR (75 MHz, CDCl_3_) δ 20.1(CH_3_), 55.6(OCH_3_), 111.5(CH), 124.3(C), 128.4(CH), 129.9(C), 136.5(CH), 159.9(C), 189.9(CHO) ppm; HRMS(EI^+^, TOF) *m*/*z* calcd for C_9_H_10_O_2_ [M]^+^ 150.06753, found 150.06790.

*2-Methoxy-3-methylbenzaldehyde* (**5b**)

Aldehyde **4b** (2.070 g, 15.20 mmol) was dissolved in DMF (9 mL) in an oil bath at 50 °C. The mixture was added K_2_CO_3_ (2.666 g, 19.29 mmol) and iodomethane (1.40 mL, 22.5 mmol), and stirred for 1 h. Upon reaching room temperature, water (20 mL) was added, and pH adjusted to 7 with 1 M HCl before extraction with ethyl acetate (2 × 10 mL). The combined organic phases were washed with brine (10 mL), dried over anhydrous MgSO_4_ and concentrated under reduced pressure. The remains were purified with flash chromatography (Petroleum ether/ethyl acetate 9/1) to afford 1.895 g (83%) of **5b** as an oil.

^1^H NMR (300 MHz, CDCl_3_) δ 2.35 (s, 3H), 3.90 (s, 3H), 7.17 (dd, *J =* 7.6, 7.6 Hz, 1H), 7.46 (ddd, *J =* 7.5, 1.7, 0.7 Hz, 1H), 7.70 (dd, *J =* 7.8, 1.7 Hz, 1H), 10.39 (d, *J =* 0.7 Hz, 1H); ^13^C NMR (75 MHz, CDCl_3_) δ 15.4(CH_3_), 63.0(OCH_3_), 124.3(CH), 126.4(CH), 129.1(C), 132.2(C), 137.5(CH), 161.7(C), 190.2(CHO) ppm; HRMS(EI^+^, TOF) *m*/*z* calcd for C_9_H_10_O_2_ [M] ^+^ 150.06753, found 150.06788.

*2-Formyl-6-methylphenyl methanesulfonate* (**5c**)

Aldehyde **4b** (2.413 g, 17.72 mmol) was dissolved in DCM (13 mL) and added triethylamine (5 mL) at 0 °C. Methanesulfonic chloride (2.812 g, 24.54 mmol) was added dropwise under continued stirring, and allowed to react for another 30 min. The reaction mixture was partitioned between EtOAc (200 mL) and saturated aqueous sodium bicarbonate (100 mL). The organic phase was washed with 3 M HCl (100 mL) and brine (100 mL), before dried over anhydrous MgSO_4_. The organic phase was then concentrated under reduced pressure to yield 2.462 g (65%) of **5c** as a thick oil. This oil was applied without further purification.

^1^H NMR (300 MHz, CDCl_3_) δ 2.46 (s, 3H), 3.38 (s, 3H), 7.36 (dd, *J =* 7.6, 7.5 Hz, 1H), 7.54 (dd, *J =* 7.5, 0.7 Hz, 1H, next to aldehyde), 7.77 (d, *J =* 7.6 Hz, 1H), 10.21 (s, 1H); ^13^C NMR (75 MHz, CDCl_3_) δ 16.7(CH_3_), 38.8(CH_3_SO_3_), 127.4(CH), 128.4(CH), 137.6(CH), 188.7(CHO) ppm (Quaternary C are not assigned due to impurities); IR (NaCl): 1700(CHO), 1351(CH_3_SO_3_), 1190(CH_3_SO_3_), 1144, 867 cm^−1^.

#### 3.2.6. Oxidation of Methylchrysene

*Chrysene-3-carboxylic acid* (**6**)

3-methylchrysene (**3b**) (50 mg, 0.21 mmol) and KMnO_4_ (100 mg, 0.50 mmol) were dissolved in pyridine/water (1 mL/2 mL). The reaction mixture was heated at reflux for 2 h, and then added more KMnO_4_ (200 mg, 1.0 mmol). The mixture was refluxed overnight. A precipitate of MnO_2_ was filtered off. The filtrate was acidified by addition of HCl and extracted with EtOAc (2 × 25 mL). The combined organic phases were dried over MgSO_4_ and concentrated under reduced pressure. The crude product obtained was purified by flash chromatography (Petroleum ether/ethyl acetate 1/4) to yield 14.6 mg (25%) of **6** as a white solid. Melting point: ˃300 °C.

^1^H-NMR (300 MHz, CD_3_OD) δ 8.51–8.62 (m, 2H), 8.95 (d, *J =* 7.8 Hz, 1H), 8.99–9.04 (m, 4H), 9.72 (d, *J =* 8.9 Hz, 1H), 9.79 (d, *J =* 8.2 Hz, 1H), 9.86 (d, *J =* 9.2 Hz, 1H), 10.30 (s, 1H), 14.08 (br. S, 1H, COOH) ppm; ^13^C NMR* (75 MHz, CD_3_OD) δ 130.6, 133.0, 133.4, 134.7, 135.7, 136.5 (2carbons), 136.7, 137.57, 137.60, 137.7, 138.0, 138.4, 138.5, 138.7, 139.4, 141.4, 143.7, 177.0 ppm; HRMS (ESI^−^, TOF) *m/z* calcd for C_19_H_11_O_2_ [M − H]^−^ 271.07645, found 271.07529.

*The ^13^C NMR was performed with 1 s extra delay between each scan.

## 4. Conclusions

We succeeded in making 1-, 2-, 3- and 6-methylchrysene (**3a**, **3d**, **3b** and **3c**, respectively) as pure compounds in gram scale. Unfortunately, 4-methylchrysene did not form from methoxy-stilbenoid **2e** in an eliminative photocyclization as previously described for the corresponding 4-methylphenanthrene. Potassium permanganate oxidation of methyl-chrysenes is not an effective reaction as it digests the whole ring system, but we were able to obtain a modest yield of chrysene-3-carboxylic acid (**6**) from 3-methylchrysene (**3b**).

## Supplementary Materials

The following are available online at https://www.mdpi.com/article/10.3390/molecules28010237/s1, ^1^H and ^13^C NMR of compounds **2a**–**f**, **3a**–**e**, **5a**–**c**, **6**.

## Sample Availability

Samples of the compounds **3a**–**e** are available from the authors.
