# Supplementary material for: Regiospecific Photochemical Synthesis of Methylchrysenes
Source: Molecules. 2022 Dec 28;28(1):237. doi: 10.3390/molecules28010237 (PMC9822284; doi:10.3390/molecules28010237)

Supplementary material

Regiospecific Photochemical Synthesis of Methylchrysenes

Thomas Böhme[1], Mari Egeland[1], Marianne Lorentzen[1], Mohamed F. Mady[2], Michelle F. Solbakk[1], Krister S. Sæbø[1], Kåre B. Jørgensen[1,*]

1) Department of Chemistry, Bioscience and Environmental Engineering, University of Stavanger, P.O. Box 8600, N-4036 Stavanger, Norway

2) Department of Chemistry and Earth Sciences, College of Arts and Sciences, Qatar University, P.O. Box 2713, Doha, Qatar

*Correspondence: kare.b.jorgensen@uis.no; Tel.: (+47) 51 83 23 06.

Contents

^{1}H and ^{13}C-NMR spectra

(*E/Z*)-1-(2-Methylstyryl)naphthalene (**2a**) S2
(*E/Z*)-1-(4-Methylstyryl)naphthalene (**2b**) S3
(*E/Z*)-1-(2-Phenylprop-1-en-1-yl)naphthalene (**2c**) S4
(*E/Z*)-1-(2-Methoxy-5-methylstyryl)naphthalene (**2d**) S5
(*E/Z*)-1-(2-Methoxy-3-methylstyryl)naphthalene (**2e**) S6
(*E/Z*)-2-Methyl-6-(2-(naphthalen-1-yl)vinyl)phenyl methanesulfonate (**2f**) S7
1-Methylchrysene (**3a**) S8
3-Methylchrysene (**3b**) S9
6-Methylchrysene (**3c**) S10
2-Methylchrysene (**3d**) S11
1-Methoxy-2-methylhrysene (**3e**) S12
2-Methoxy-5-methylbenzaldehyde (**5a**) S13
2-Methoxy-3-methylbenzaldehyde (**5b**) S14
2-Formyl-6-methylphenyl methanesulfonate (**5c**) S15
Chrysene-3-carboxylic acid (**6**) S16

(*E/Z*)-1-(2-Methylstyryl)naphthalene (2a)

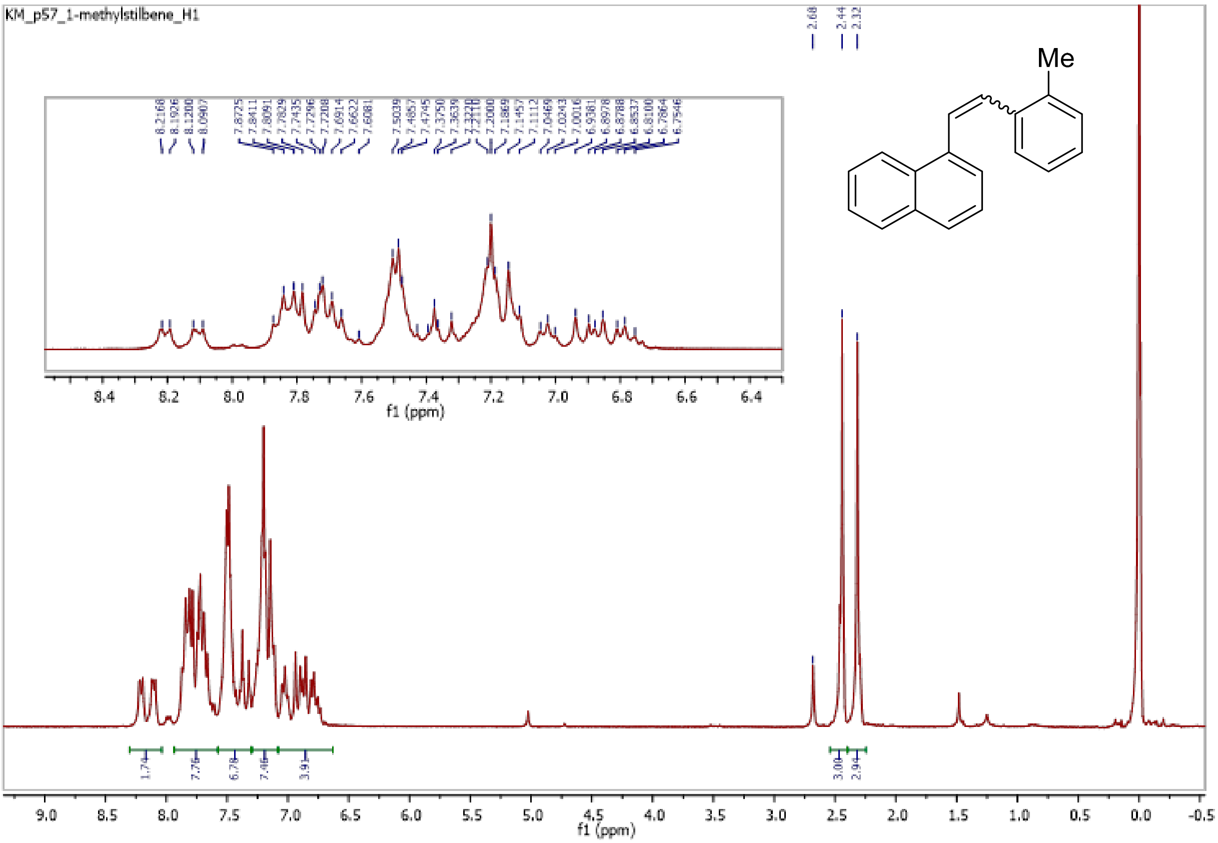

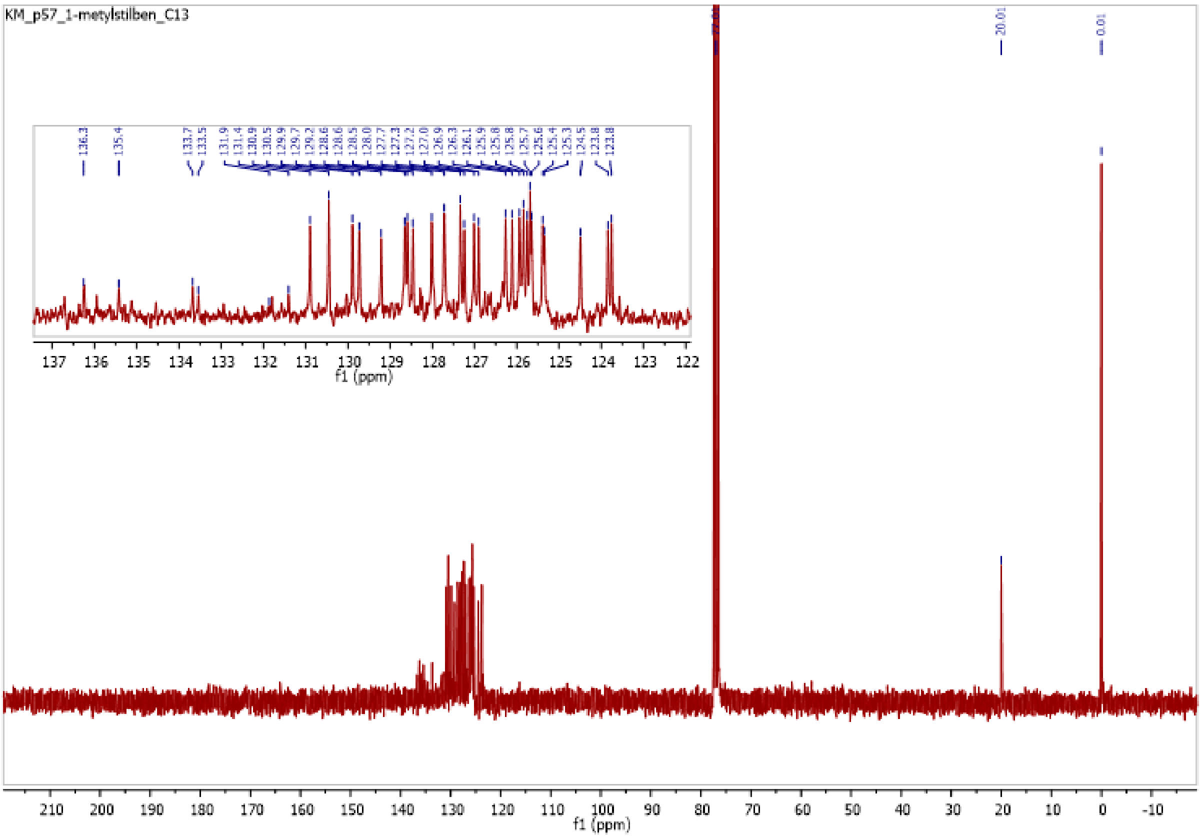

(*E/Z*)-1-(4-Methylstyryl)naphthalene (2b)

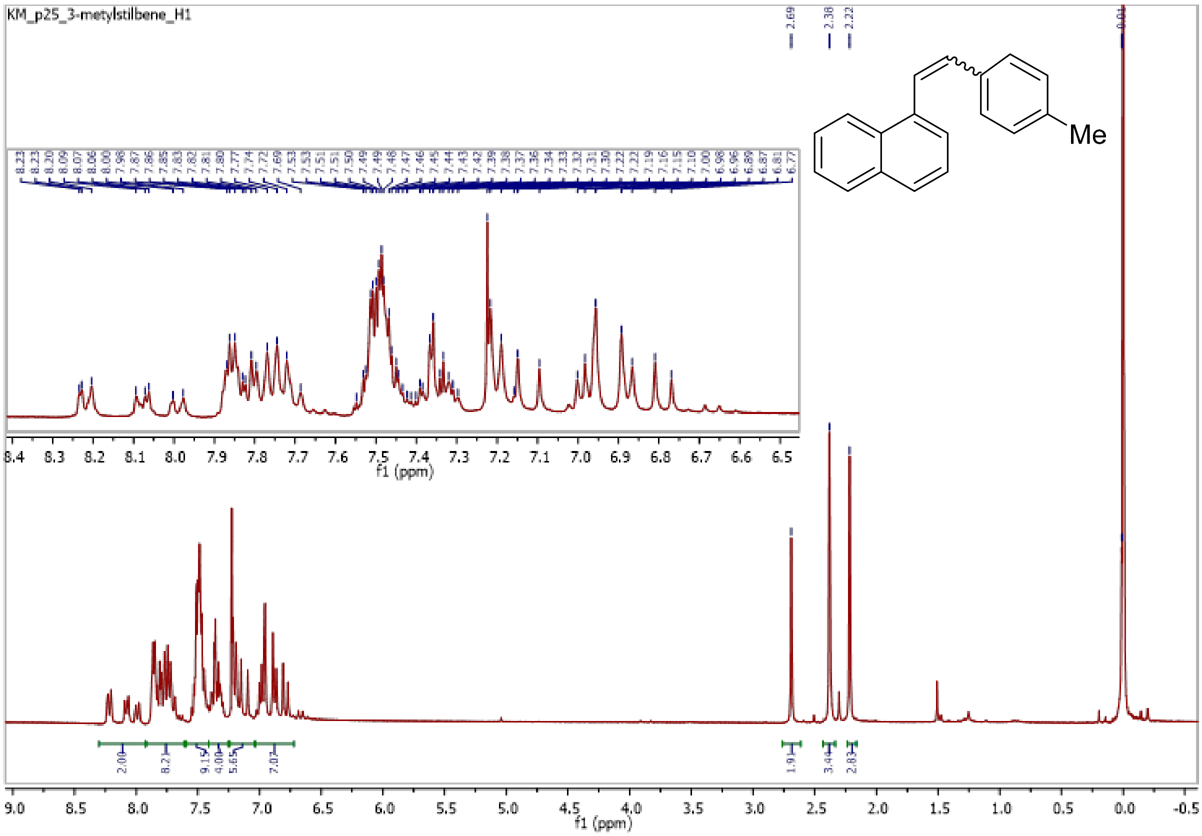

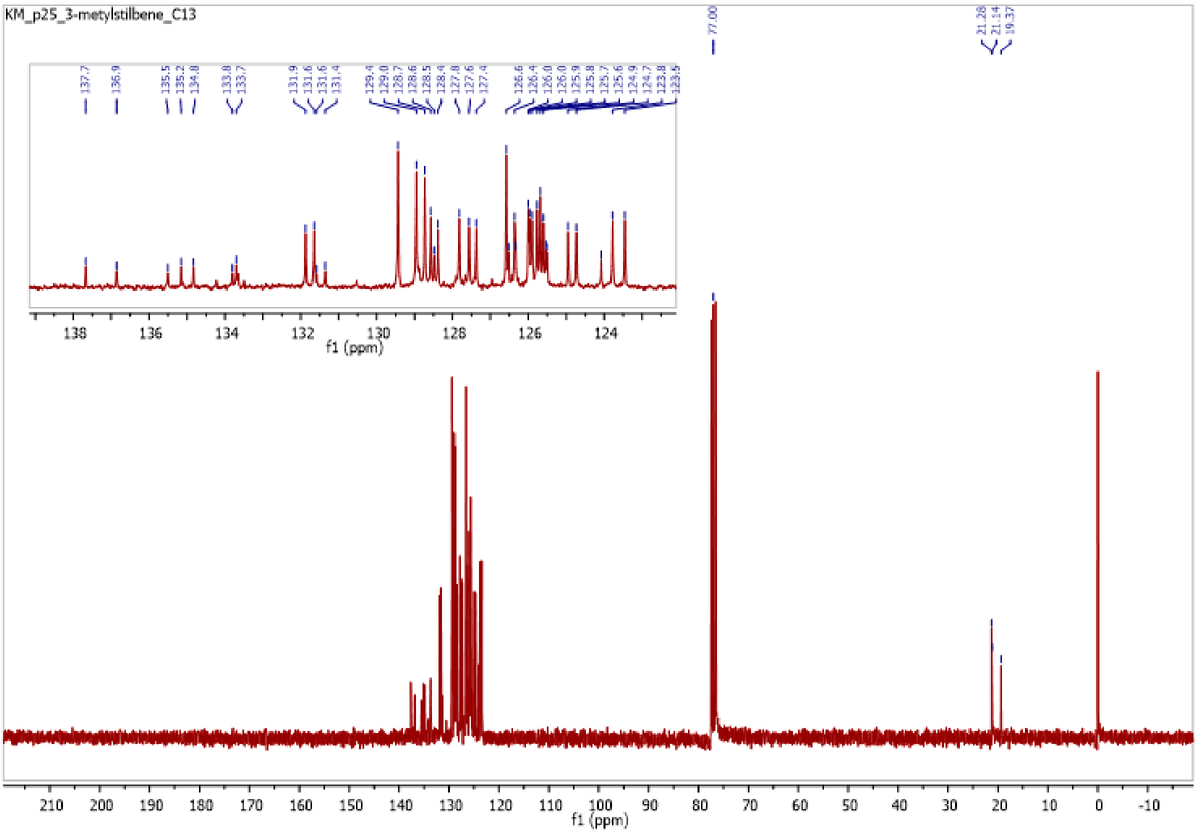

(*E/Z*)-1-(2-Phenylprop-1-en-1-yl)naphthalene (2c)

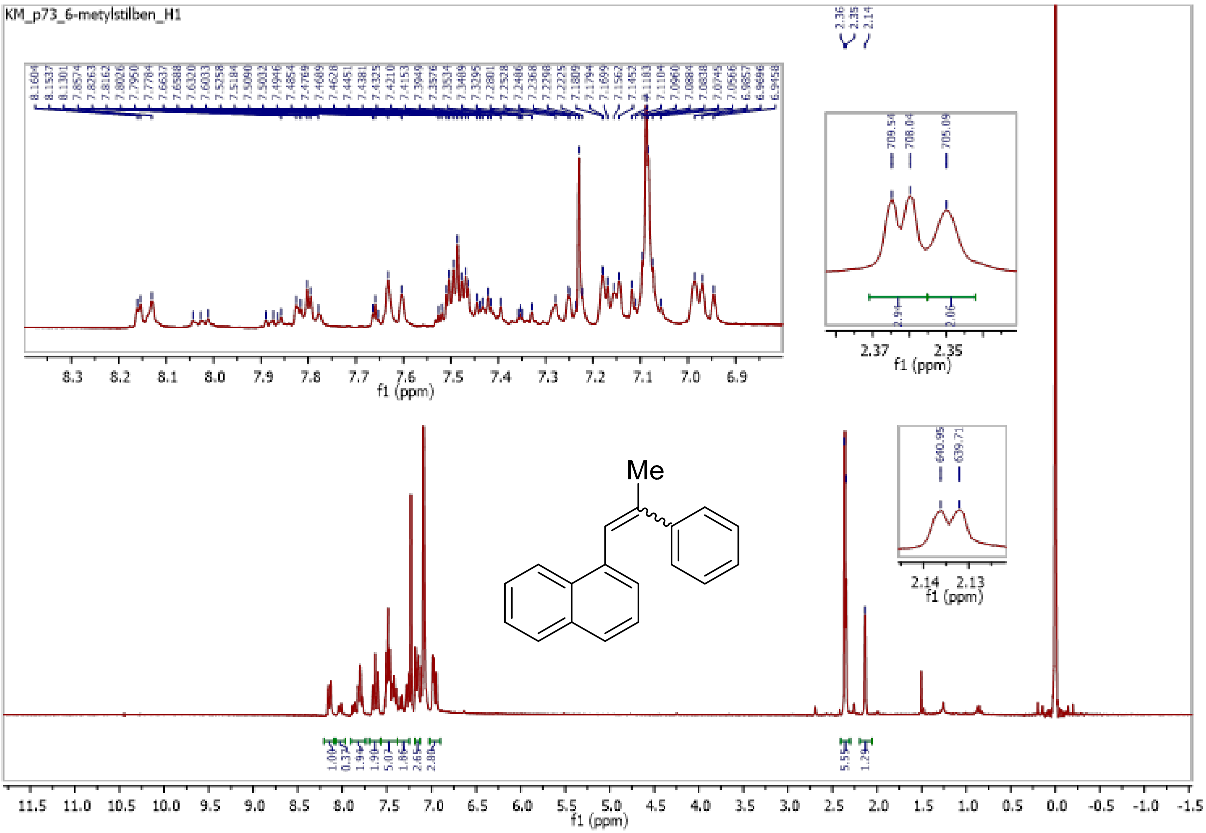

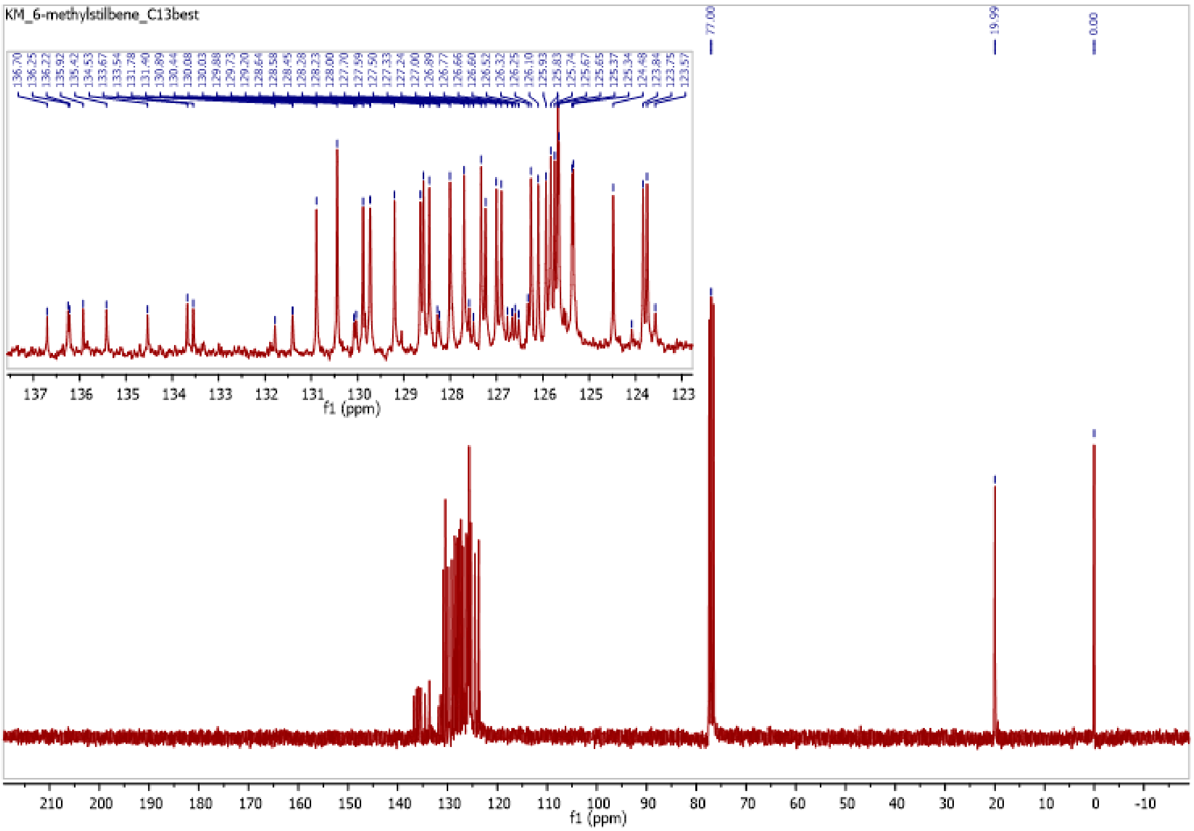

(*E/Z*)-1-(2-Methoxy-5-methylstyryl)naphthalene (2d)

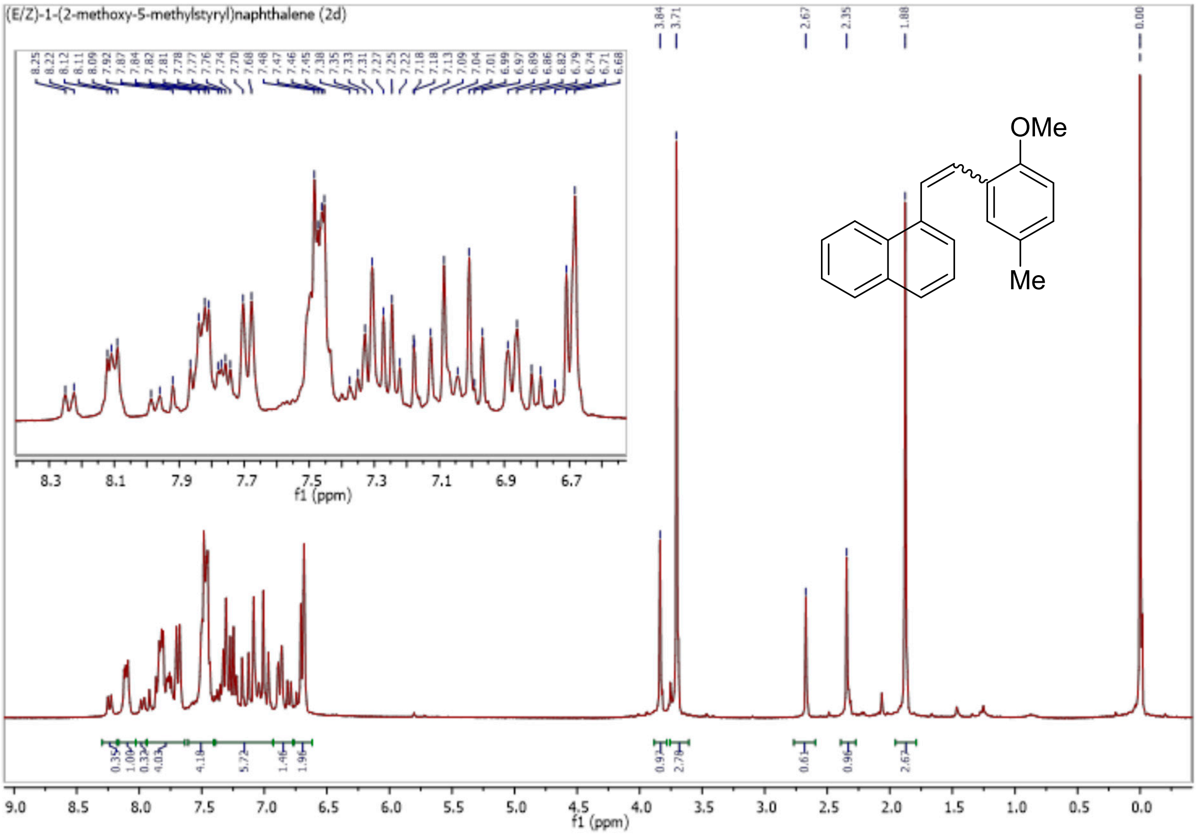

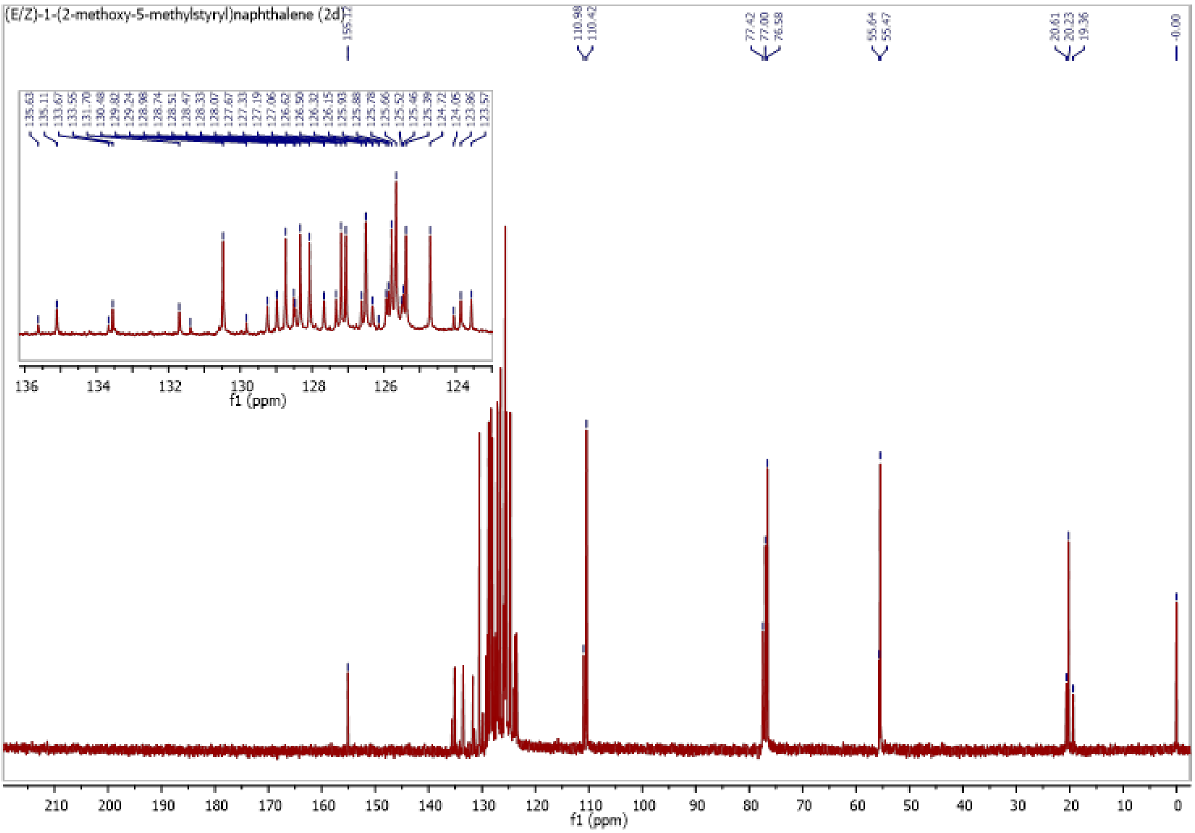

(*E/Z*)-1-(2-Methoxy-3-methylstyryl)naphthalene (2e)

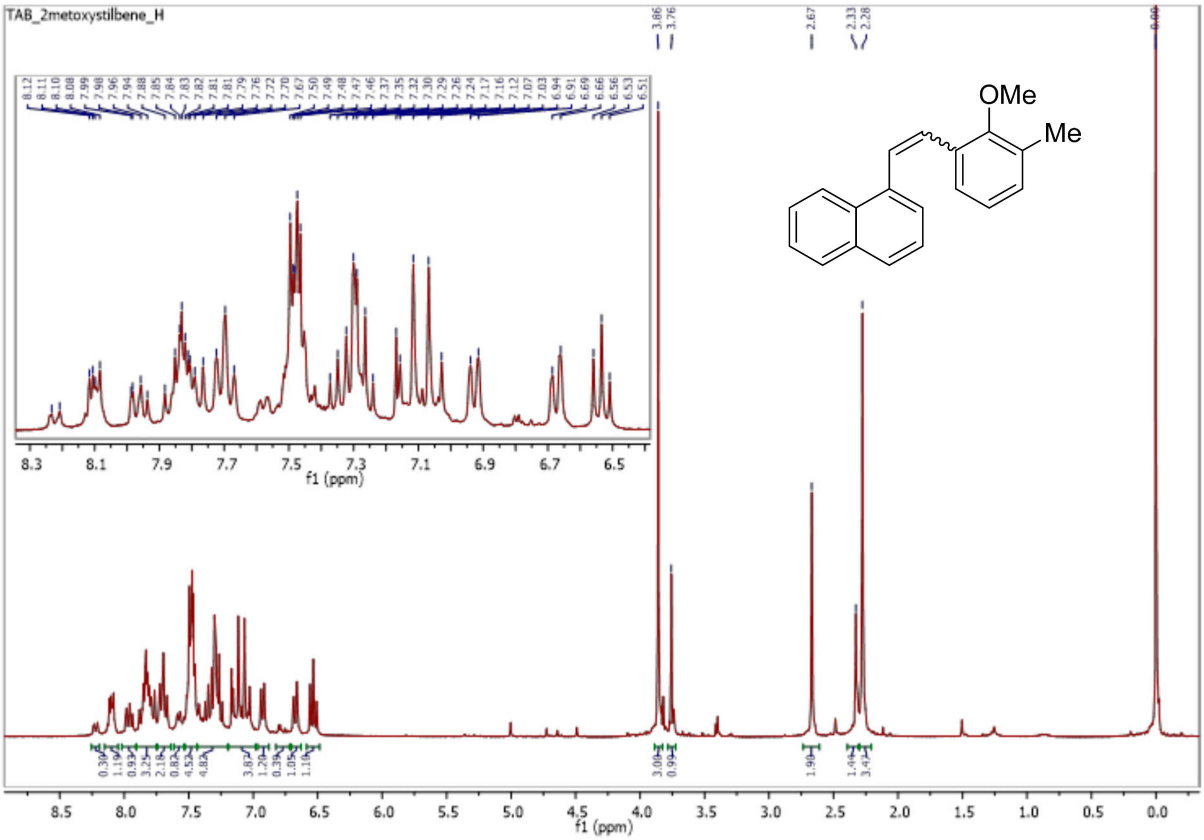

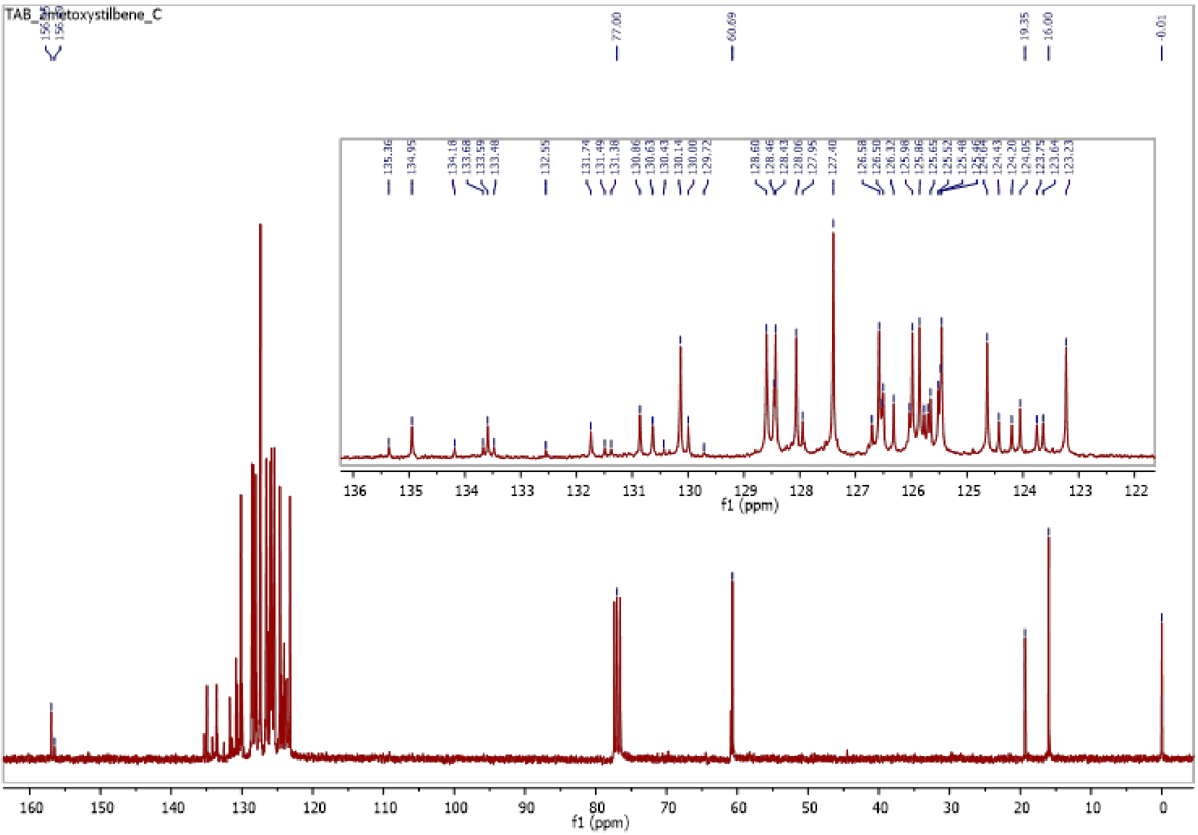

(*E/Z*)-2-Methyl-6-(2-(naphthalen-1-yl)vinyl)phenyl methanesulfonate (2f)

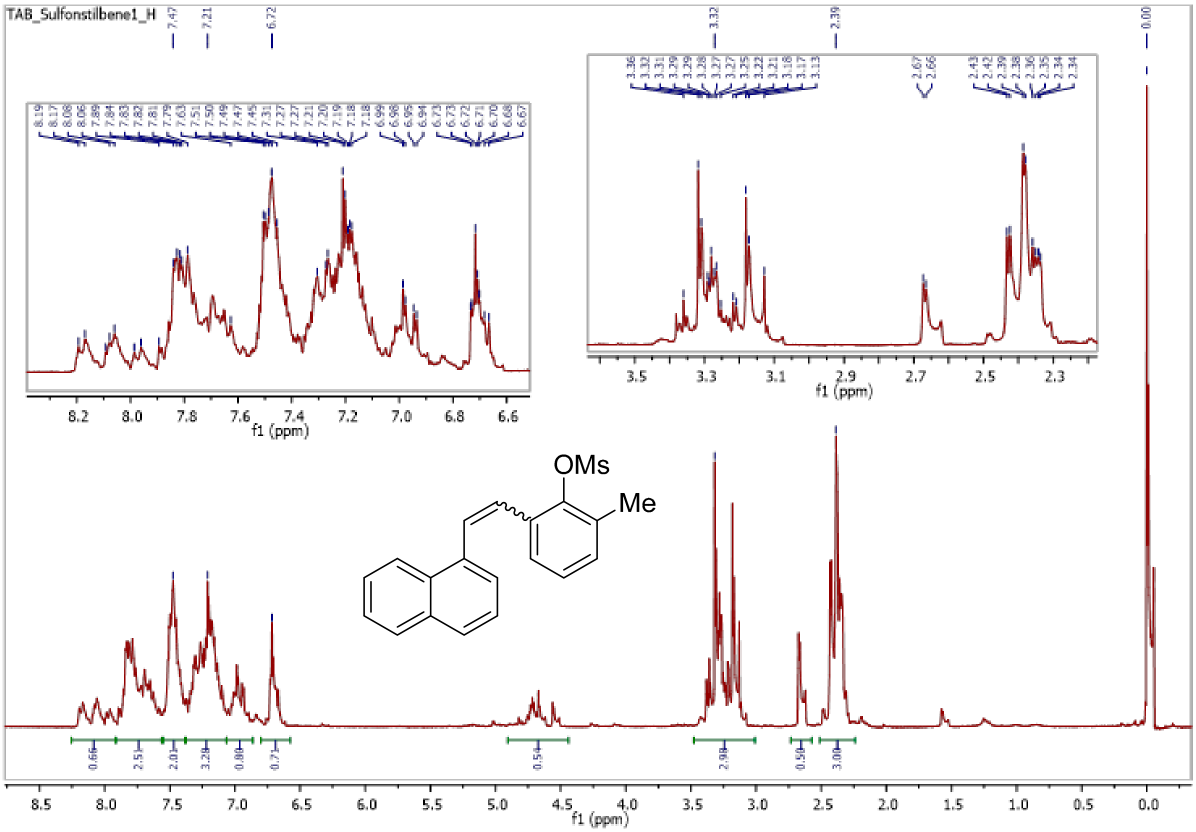

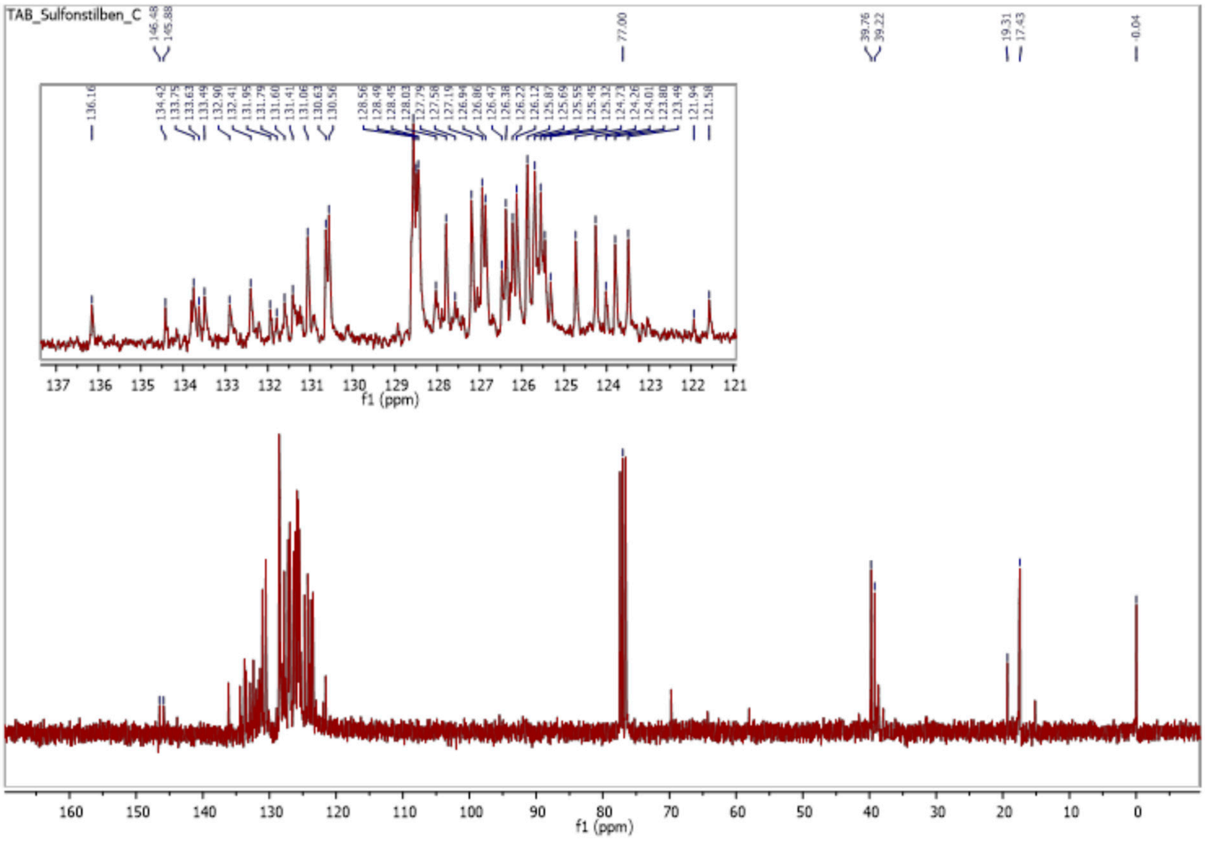

1-Methylchrysene (3a):

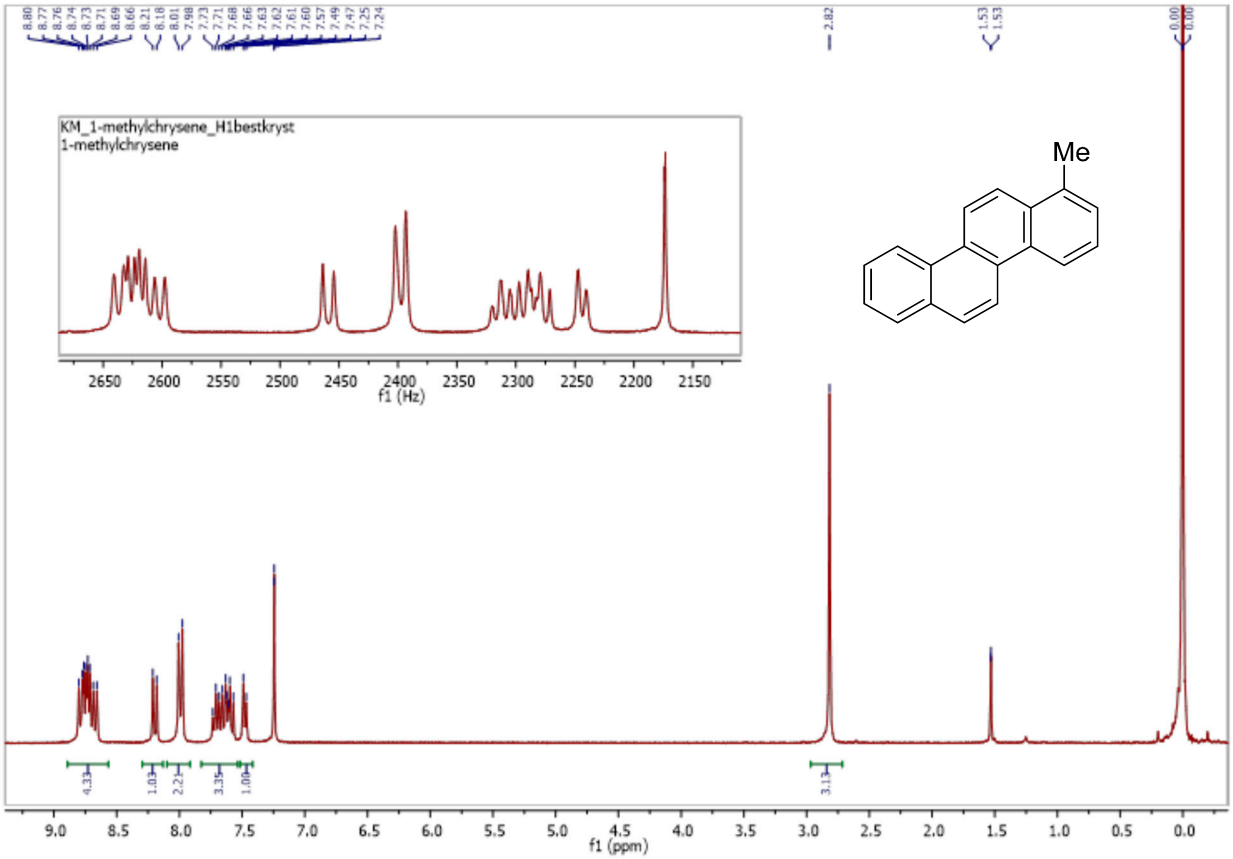

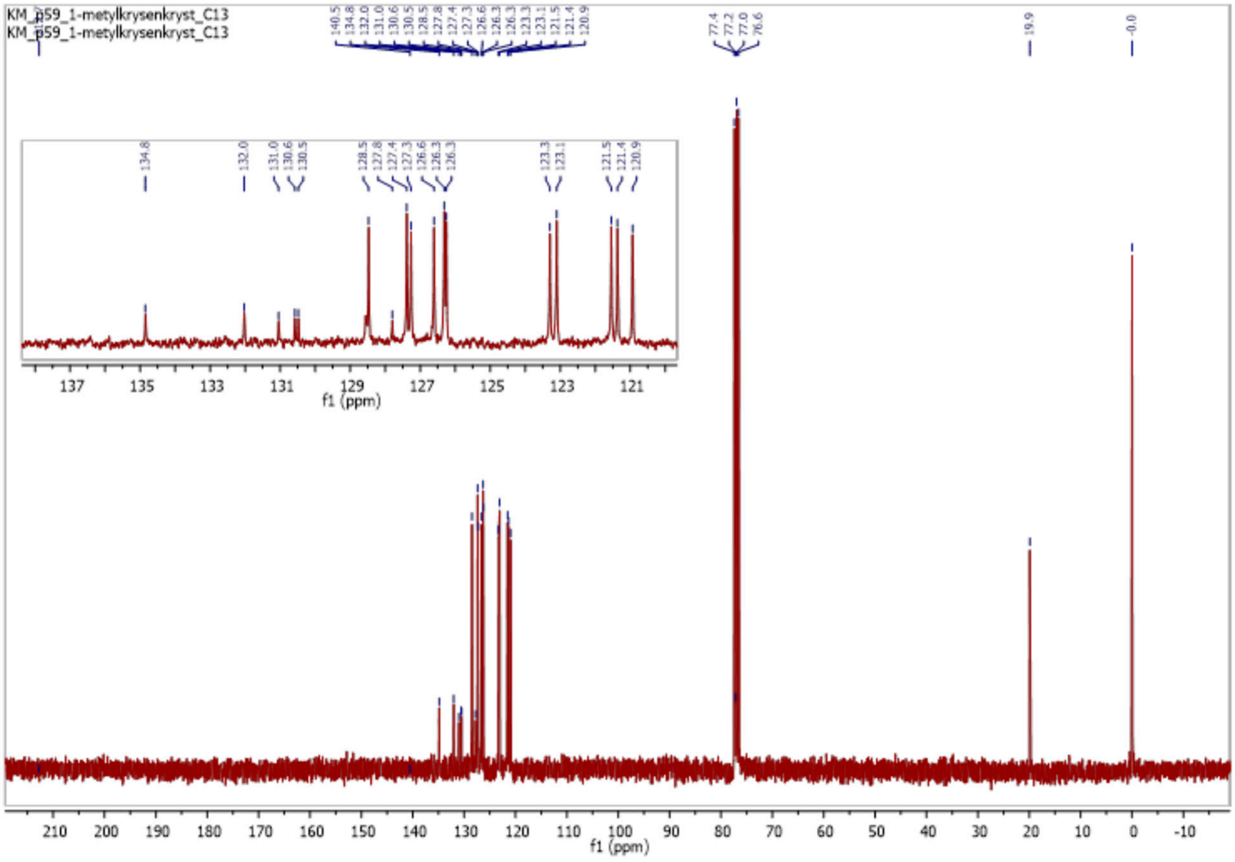

3-Methylchrysene (3b):

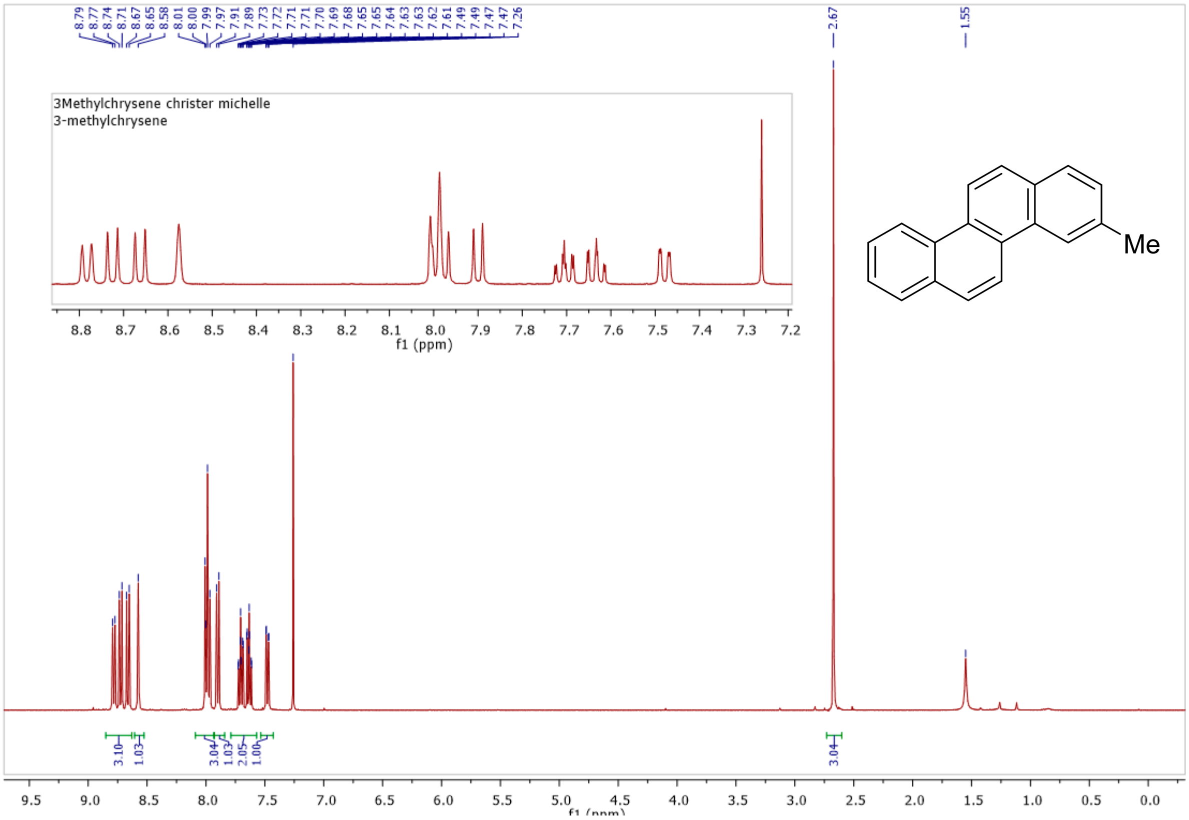

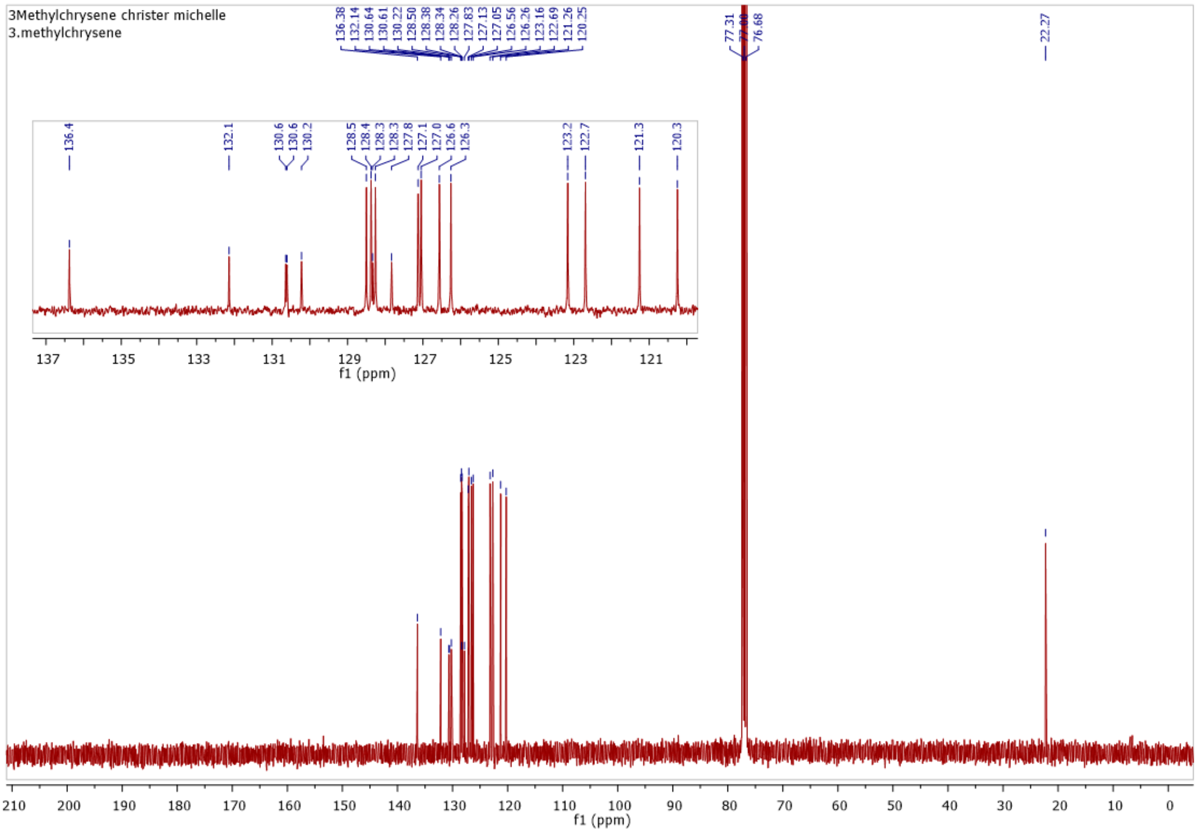

6-Methylchrysene (3c):

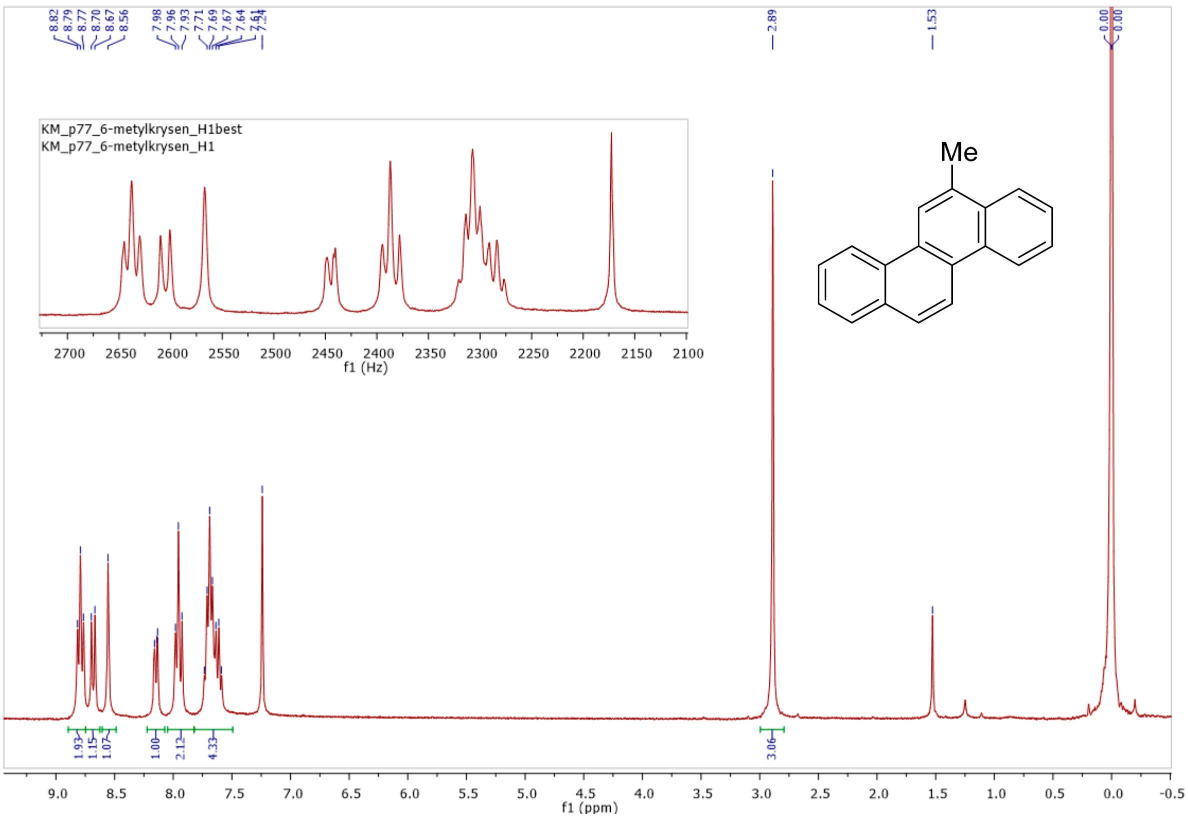

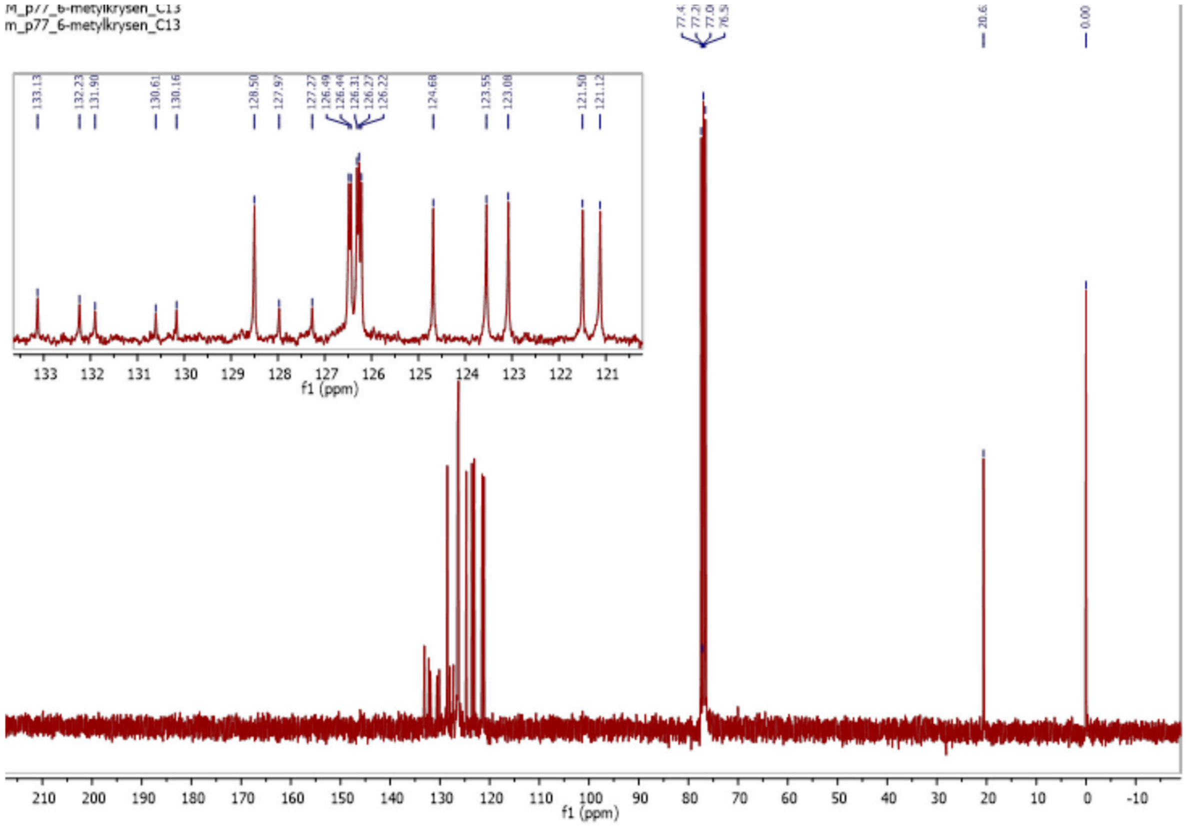

2-Methylchrysene (3d):

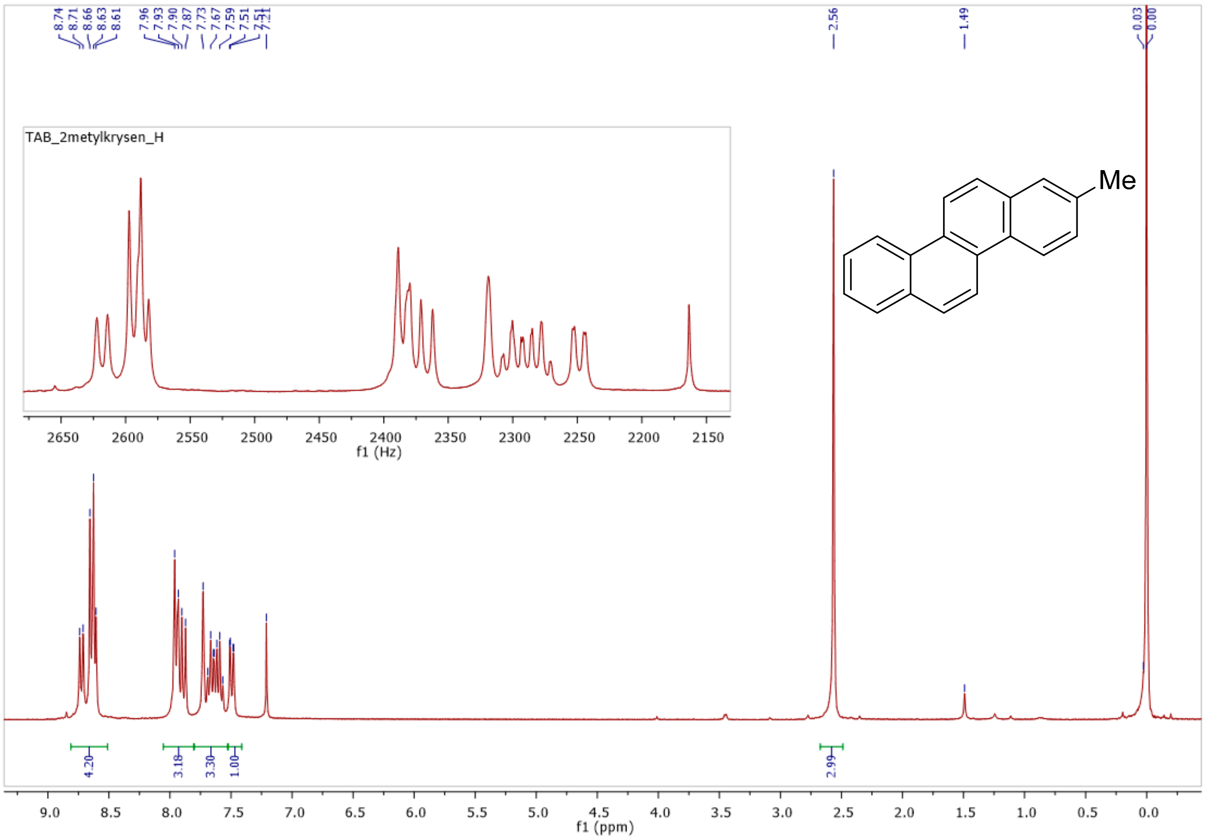

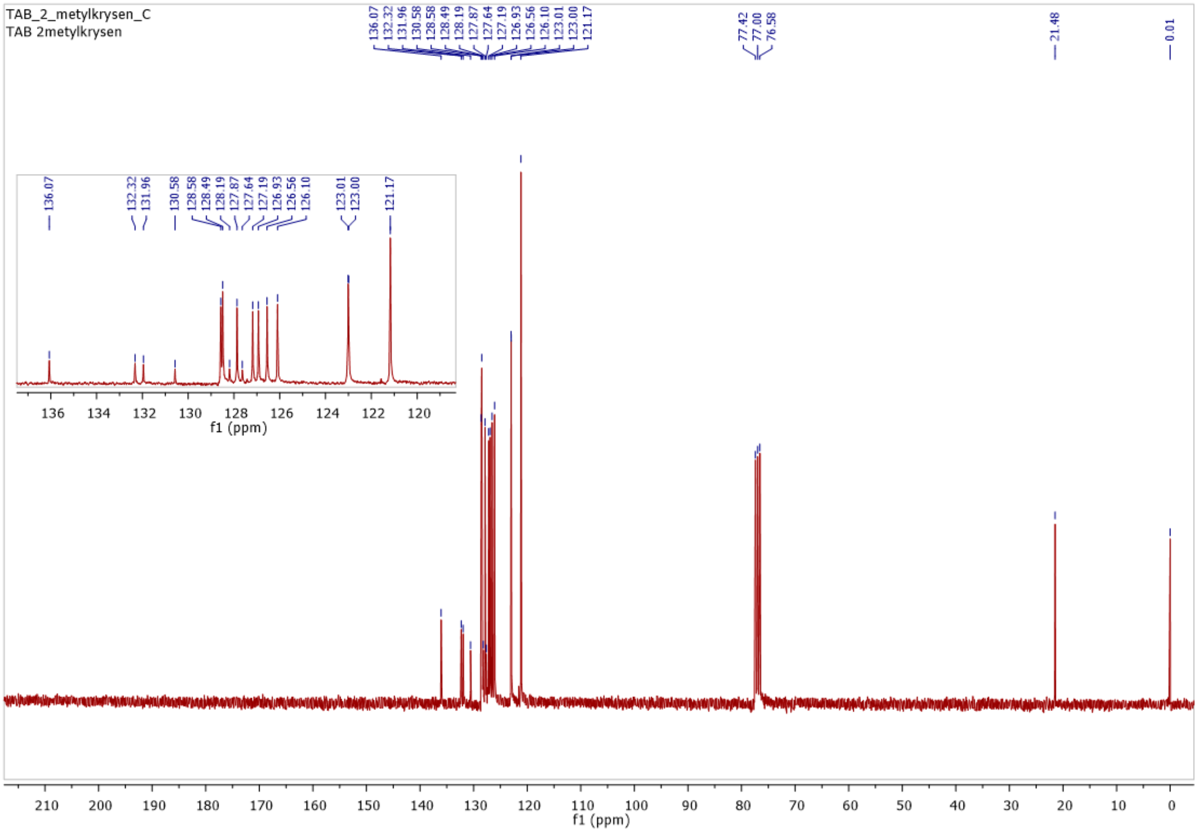

1-Methoxy-2-methylhrysene (3e)

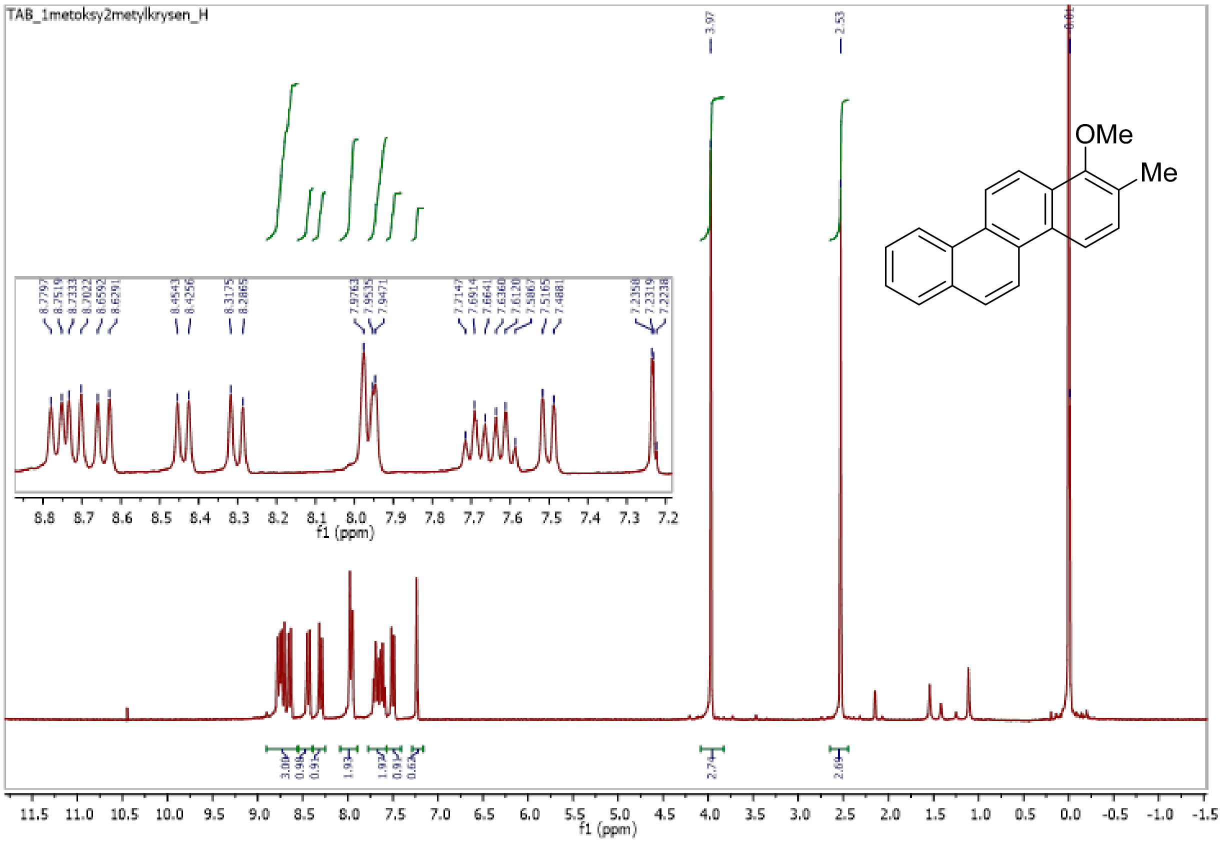

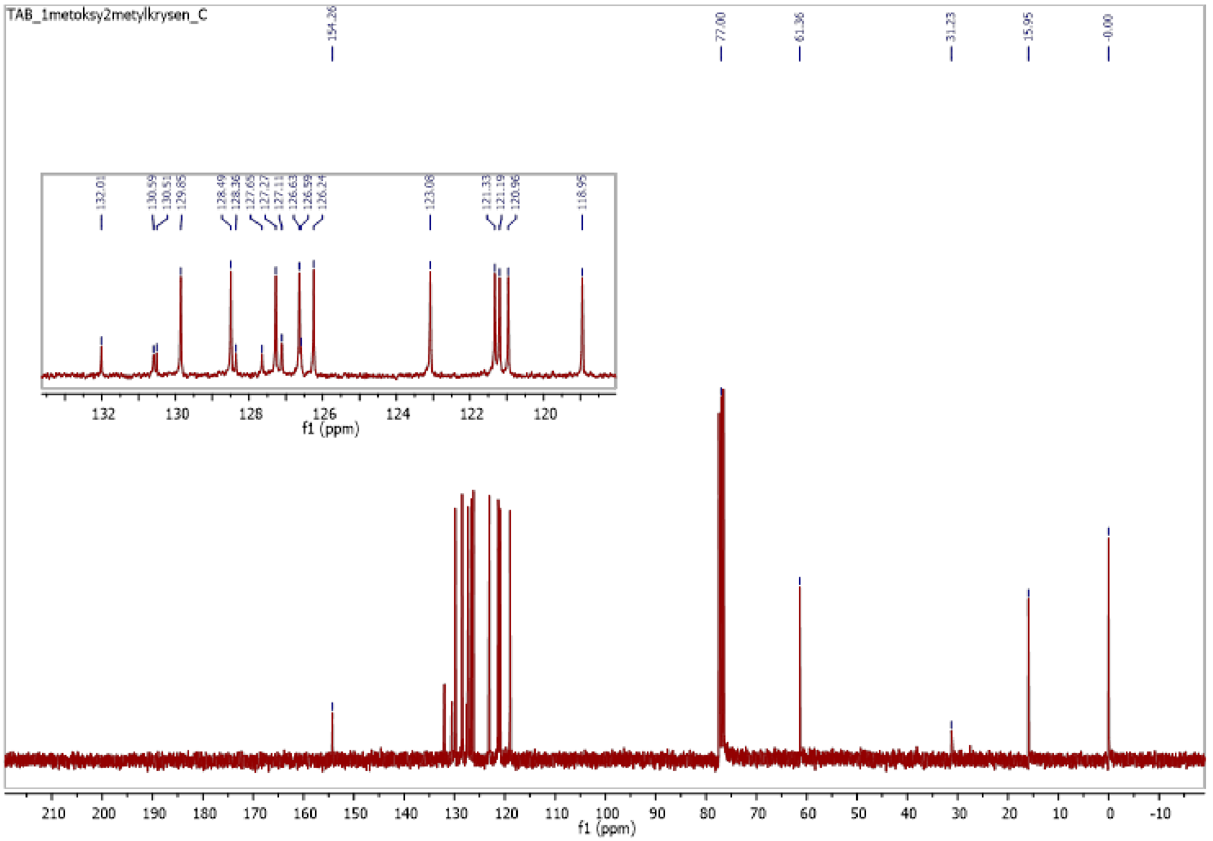

2-Methoxy-5-methylbenzaldehyde (5a)

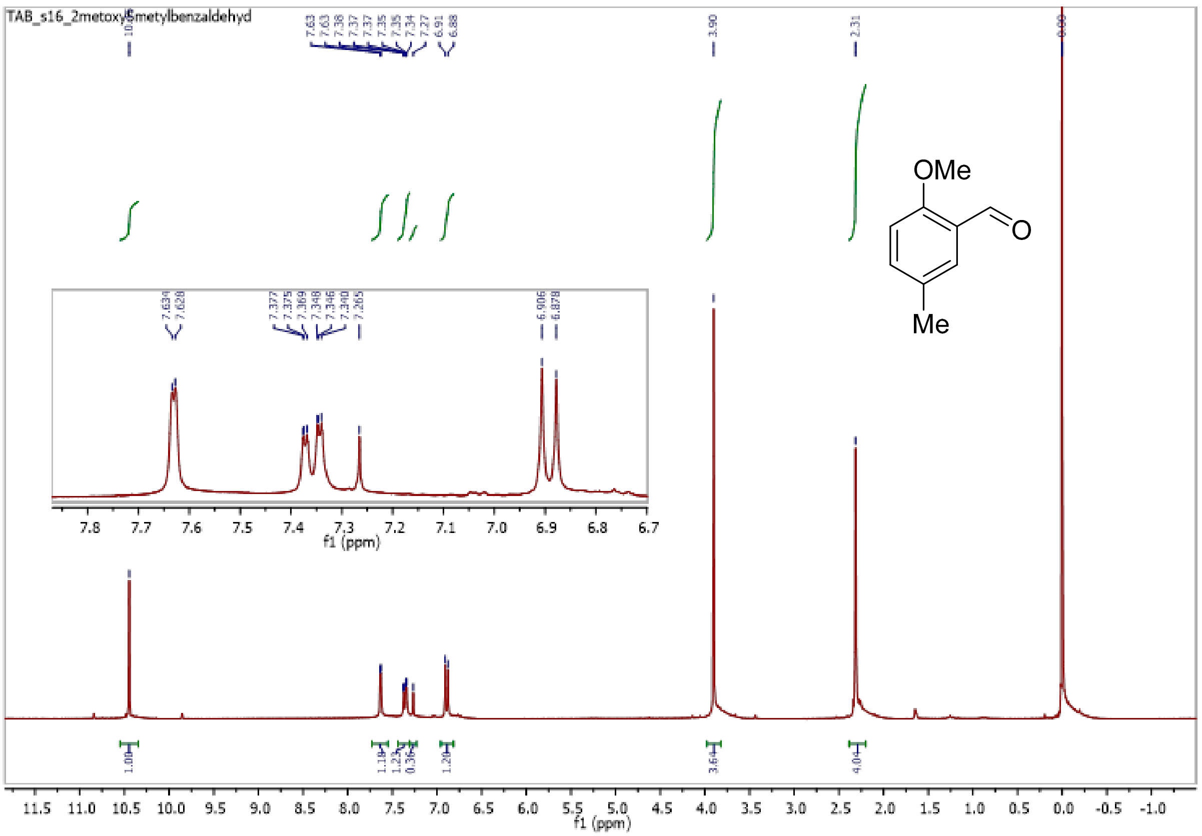

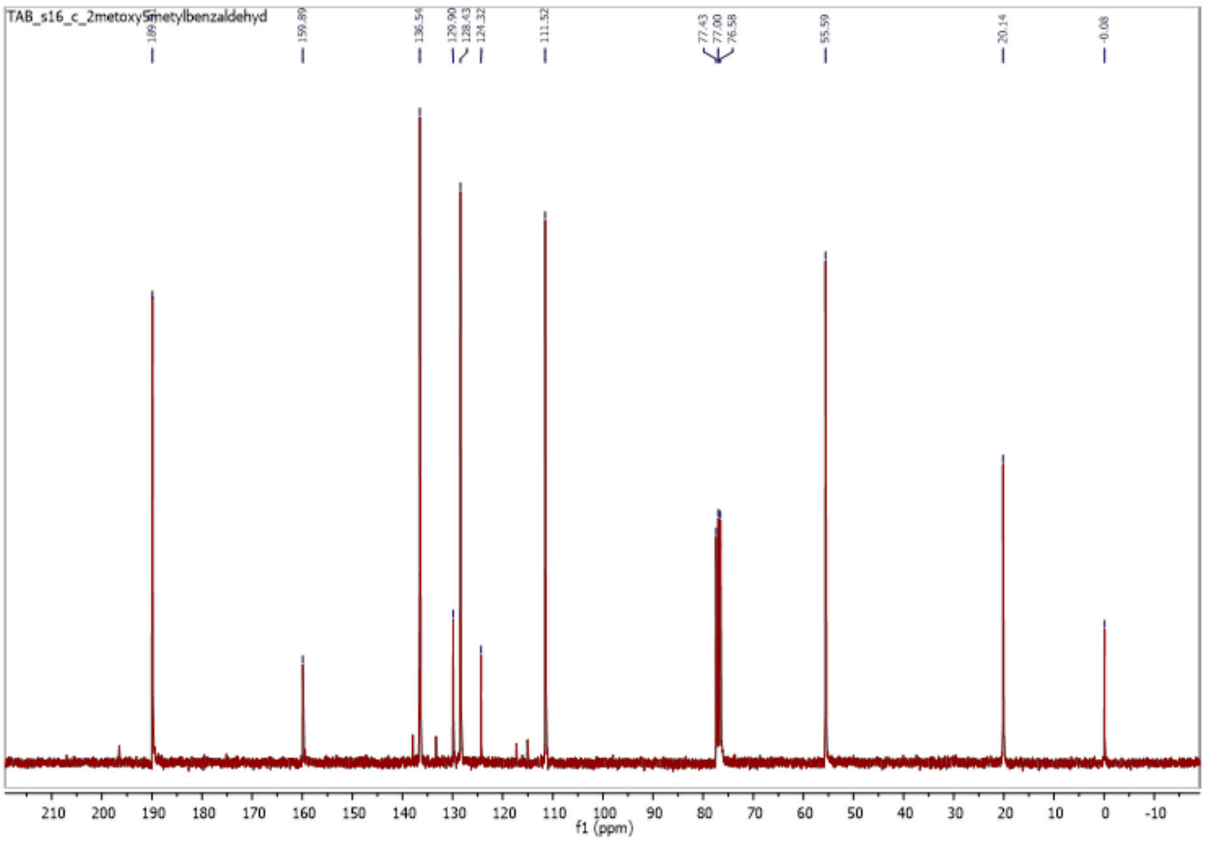

2-Methoxy-3-methylbenzaldehyde (5b)

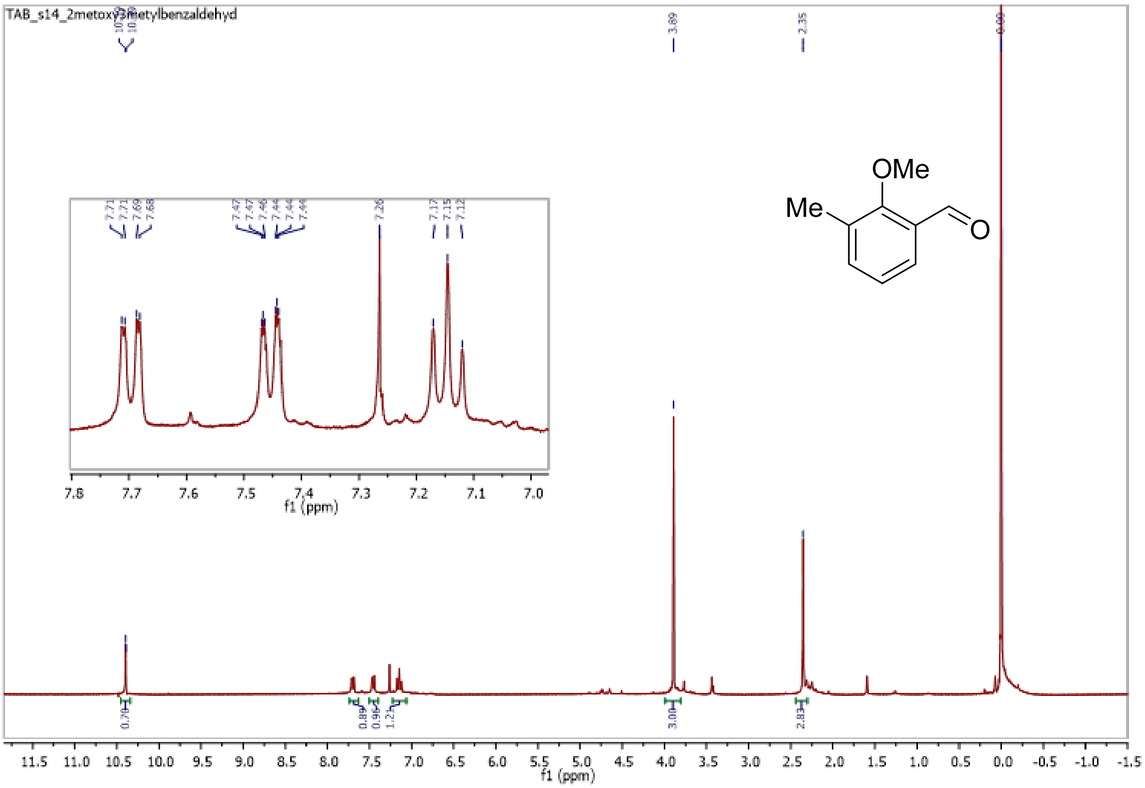

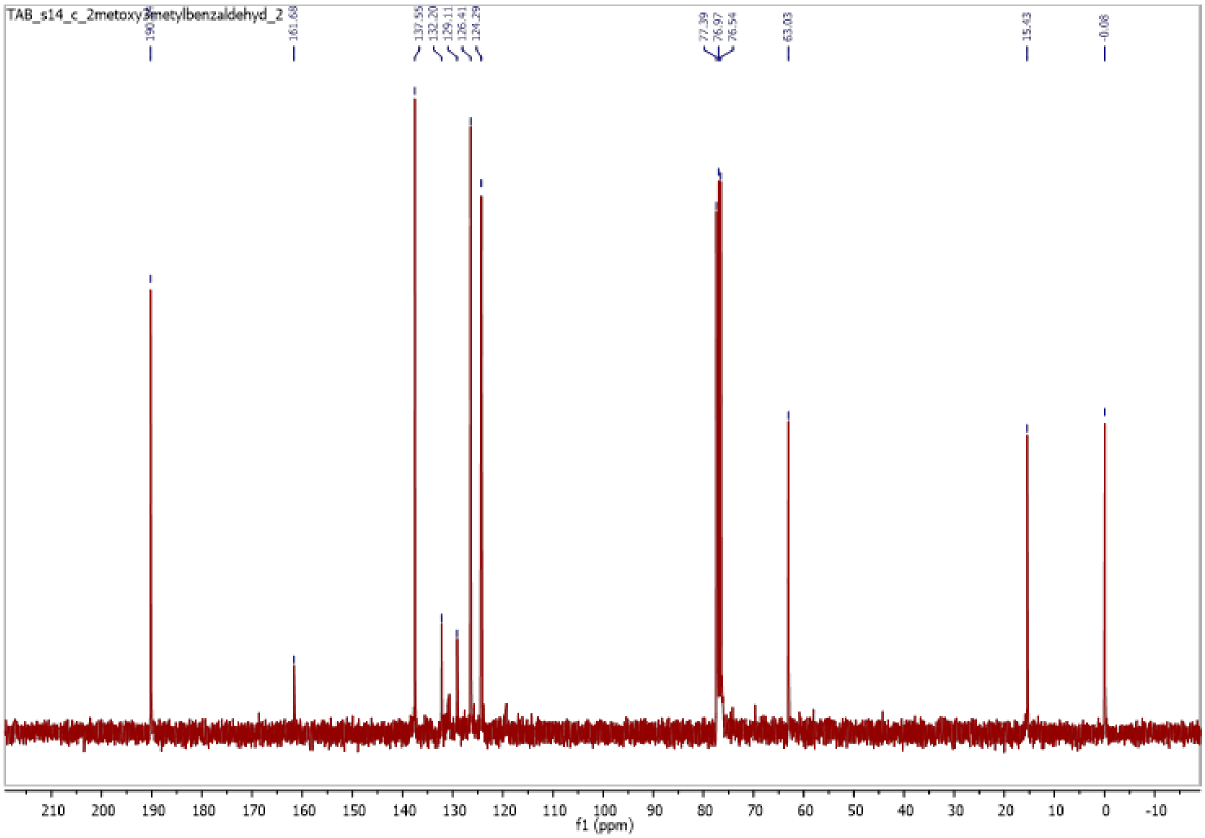

2-Formyl-6-methylphenyl methanesulfonate (5c)

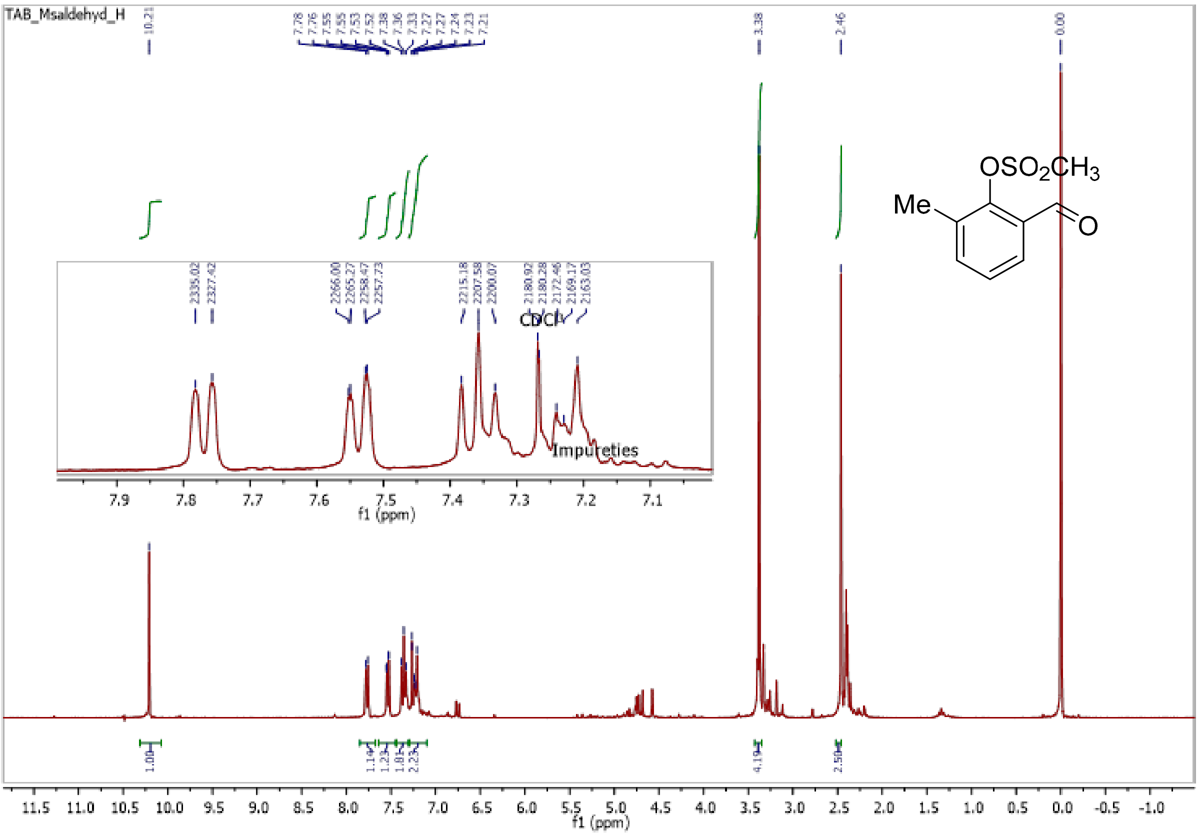

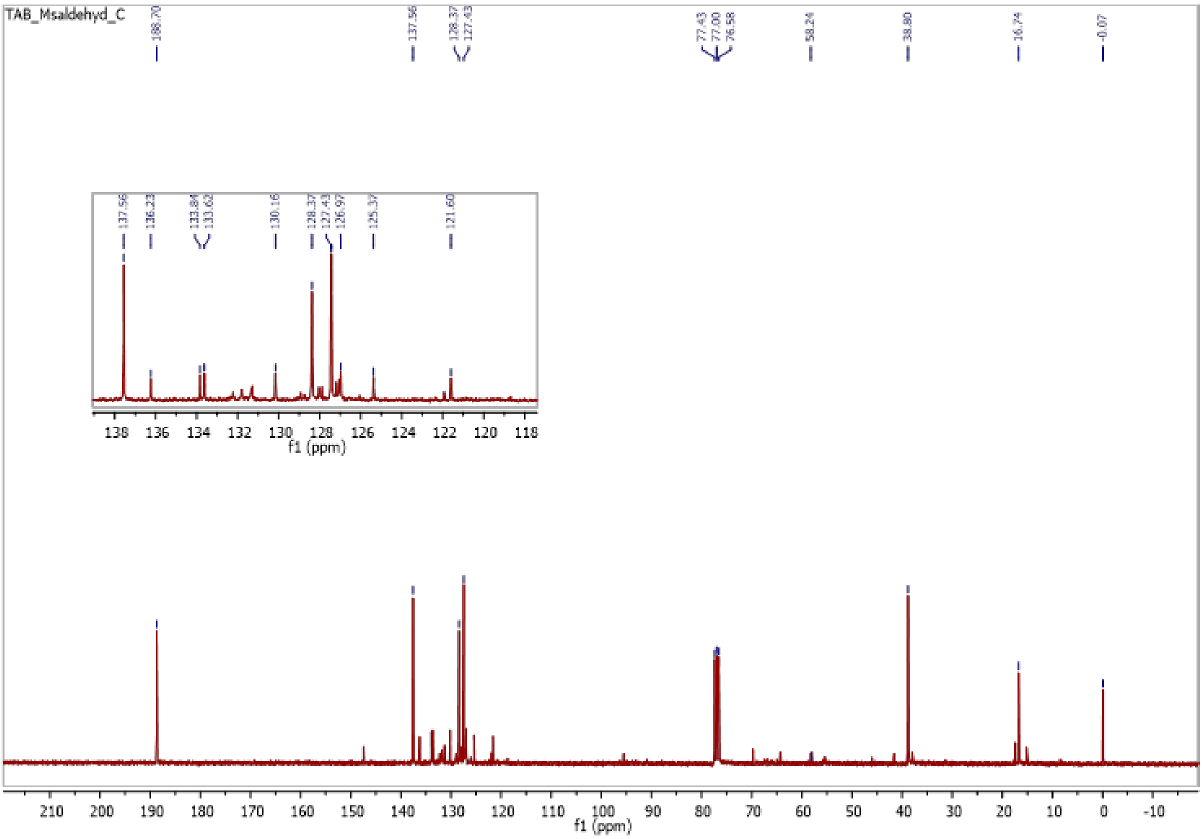

Chrysene-3-carboxylic acid (6)

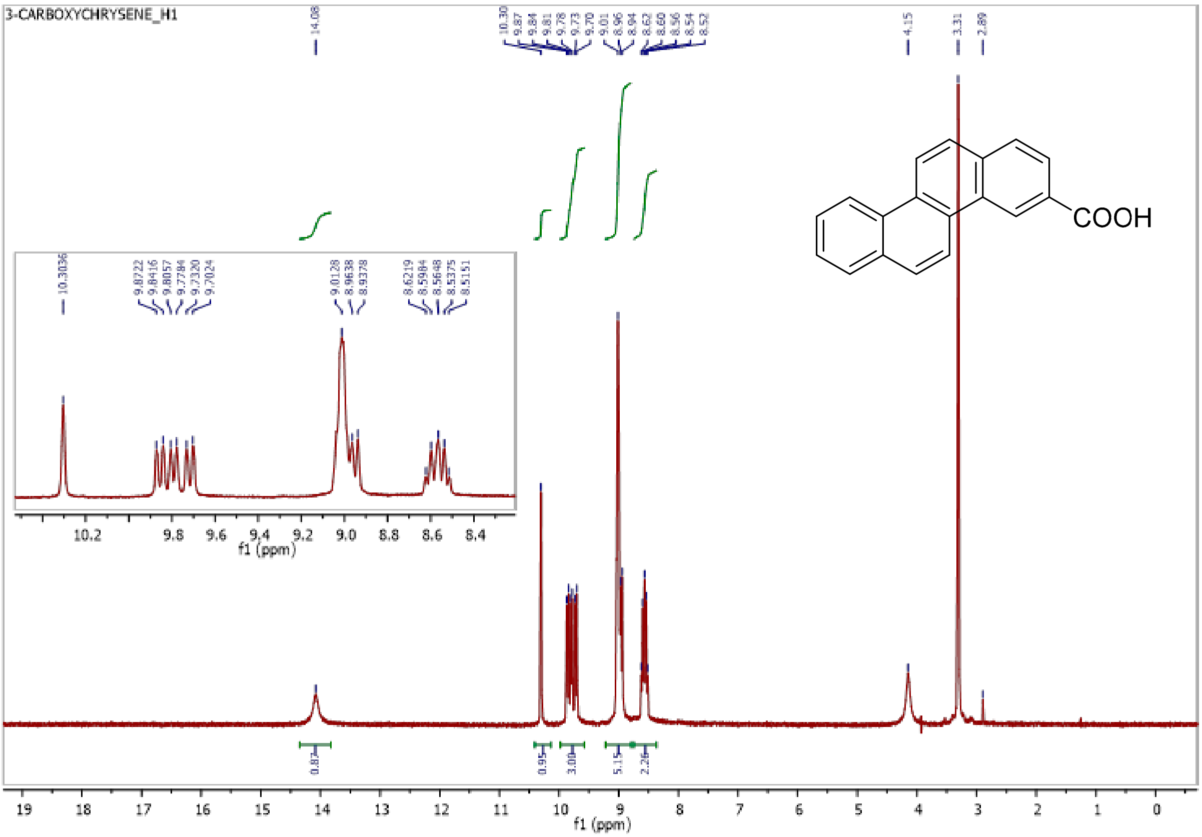

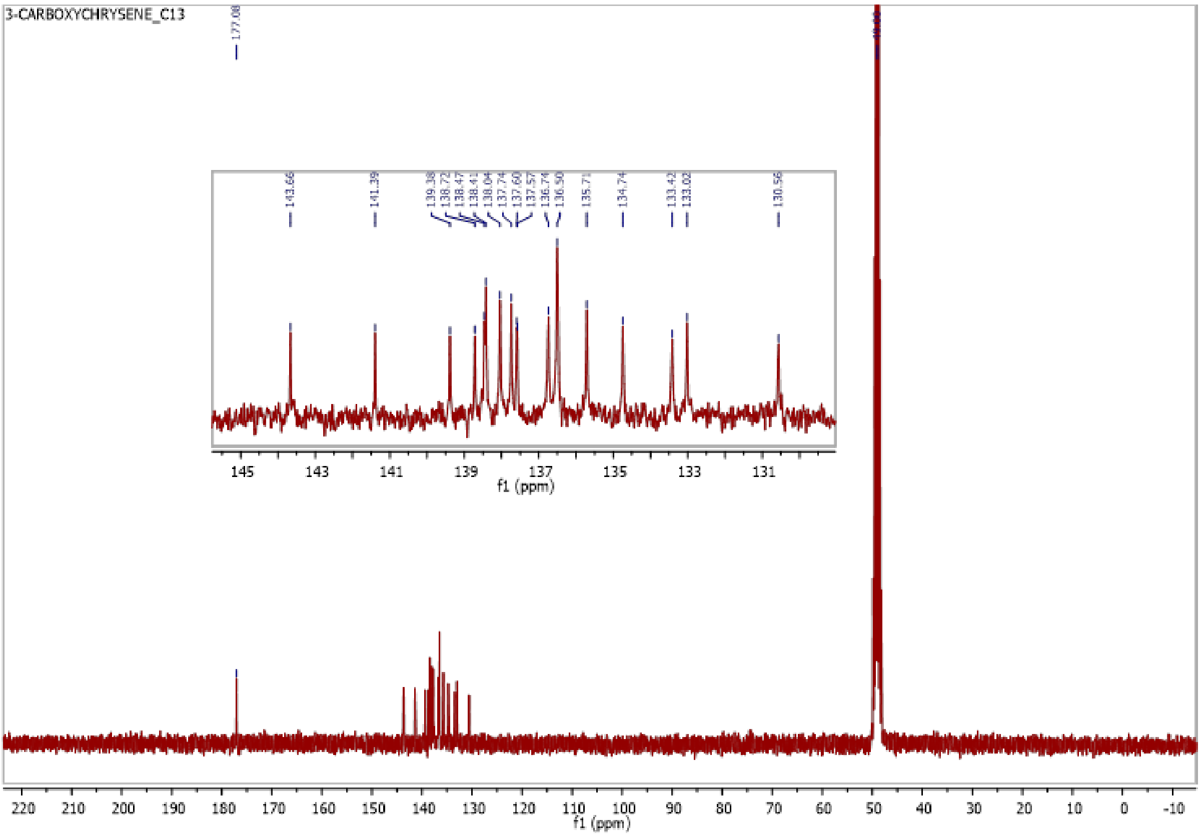

Supplement: Supplementary file 1 [file molecules-28-00237-s001.zip › molecules-2103769-supplementary.pdf]
